# Synthetic transcription factors establish the function of nine amino acid transactivation domains of *Komagataella phaffii* Mxr1

**DOI:** 10.1016/j.jbc.2025.108211

**Published:** 2025-01-22

**Authors:** Prachi Priya, Vedanth Bellad Shivashankar, Pundi N. Rangarajan

**Affiliations:** Department of Biochemistry, Indian Institute of Science, Bangalore, India

**Keywords:** transactivation, 9aaTAD, synthetic transcription factor, *Komagataella phaffii*, *Pichia pastoris*, one-carbon metabolism, Mxr1, transcription factor, zinc finger, yeast metabolism

## Abstract

The zinc finger (ZF) transcription factor Mxr1 (methanol expression regulator 1) of the methylotrophic yeast *Komagataella phaffii* (formerly *Pichia pastoris*) harbors a DNA-binding domain consisting of two C_2_H_2_ ZFs (Mxr1ZF) between amino acids 36 and 101 and a previously identified nine amino acid transactivation domain (9aaTAD) between residues 365 and 373 (TAD A, QELESSLNA). Beyond this, 21 putative 9aaTADs (designated TAD B–V) located between amino acids 401 and 1155 remain to be characterized. Here, we demonstrate that a compact synthetic transcription factor composed of Mxr1ZF and three tandem copies of TAD A can activate the transcription of Mxr1 target genes for ethanol and methanol metabolism with specificity and efficiency comparable to the full-length protein. Expression of individual synthetic transcription factors containing Mxr1ZF and each of the 20 putative 9aaTADs in *K. phaffii Δmxr1* strain revealed that 10 of these putative TADs are functional, capable of reversing the growth defect of the mutant and activating transcription of target genes required for ethanol and methanol metabolism. Functional analysis indicates that Mxr1 9aaTADs rely on General Control Nondepressible 5 (Gcn5), a histone acetyltransferase, for transactivation. These findings suggest that recruitment of Gcn5-mediated histone acetylation at target promoters is a critical step in transcriptional activation by Mxr1 9aaTADs. This study represents the first comprehensive characterization of 9aaTADs in a *K. phaffii* ZF transcription factor, providing insights into their mechanism and potential applications in synthetic biology.

The methylotrophic yeast, *Komagataella phaffii* (formerly known as *Pichia pastoris*), harbors a methanol utilization pathway comprising several methanol-inducible enzymes, such as alcohol oxidase 1 (AOX1), dihydroxyacetone synthase (DHAS1), formate dehydrogenase (FDH), and formaldehyde dehydrogenase (FLD). Amongst these, the methanol-inducible promoter of *AOX1* (*P*_*AOX1*_) is widely used for the commercial production of several recombinant proteins of commercial interest, such as vaccine antigens, therapeutic proteins, food additives, and industrial enzymes (1). License-free *K. phaffii* strains have been made available recently (2). High-density cultivation using low-cost media, ease of genetic manipulations, stable genetic constructs, efficient secretion of recombinant proteins, and post-translational modification of proteins render *K. phaffii* a highly popular eukaryotic expression system (3, 4, 5). Production of recombinant proteins from *P*_*AOX1*_ involves culturing recombinant *K. phaffii* in a medium containing glucose or glycerol as the sole source of carbon to generate high biomass and then shifting to a medium containing methanol for the induction of recombinant protein production (6). Transcription of *AOX1*, as well as several other genes of methanol metabolism, is primarily regulated by a plethora of zinc finger (ZF) transcription factors, such as Mxr1 (methanol expression regulator 1), Trm1, Mit1, Rop1, Mig1, Mig2, and Nrg1 (7, 8, 9, 10, 11, 12, 13, 14, 15, 16, 17, 18, 19). Amongst these, Mxr1 (National Center for Biotechnology Information accession number: XP_002493922.1) functions as a global regulator of multiple metabolic pathways, regulating the transcription of genes encoding key enzymes required for the metabolism of not only methanol but several other carbon compounds, such as acetate, amino acids, ethanol, and fatty acids (7, 8, 12, 13, 14). In the last 2 decades, knowledge gained from the study of transcription factors, such as Mxr1, Mit1, Mig1, and Mig2, and their DNA-binding properties has been exploited for the development of novel and methanol-free *K. phaffii P*_*AOX1*_–based expression systems (18, 20, 21, 22).

Transcription factors minimally contain two functional domains, namely the DNA-binding domain for binding to specific DNA sequences in the promoters of the target genes and the transactivation domain (TAD) for interaction with components of the transcriptional machinery, such as histone acetyl transferases, nucleosome-remodeling complexes, and general transcription factors. They also possess nuclear localization signal(s) (NLS) for their entry into the nucleus (23). *K. phaffii* Mxr1 comprises 1155 amino acids, and the DNA-binding domain consisting of two C_2_H_2_ ZFs (Mxr1ZF) is present between amino acids 36 and 101. Mxr1ZF binds to Mxr1 response elements present in the promoters of target genes such as *ALD6-1* encoding an aldehyde dehydrogenase required for ethanol metabolism, *ACS1* encoding acetyl CoA synthase 1 required for acetate metabolism, *AOX1* encoding alcohol oxidase 1 required for methanol metabolism, and genes encoding several key enzymes essential for amino acid metabolism (7, 8, 9, 12, 13, 14). An NLS is present between amino acids 75 and 85, and mutation of basic amino acid residues of NLS to alanine abrogates nuclear localization of Mxr1 (14). Several eukaryotic transcription factors contain TADs known as 9aaTADs (24, 25, 26). In Mxr1, the sequence QELESSLNA, spanning amino acids 365 to 373, functions as the 9aaTAD (14). This TAD is referred to as TAD A in this study. A truncated Mxr1 harboring 400 N-terminal amino acids (Mxr1N400) activates the transcription of *ALD6-1* and reverses the growth defect of *Δmxr1* during ethanol metabolism (14). Deletion of TAD A in Mxr1N400 (Mxr1N400ΔTAD) abrogates its ability to activate *ALD6-1* transcription. The role of TAD A in the activation of Mxr1 target genes of other metabolic pathways is not known. In addition to TAD A, Mxr1 harbors several putative 9aaTADs between amino acids 401 and 1155 (14), and determination of their role in the transactivation of Mxr1 target genes involved in the metabolism of ethanol, methanol, acetate, and amino acids remains a challenge. Here, we show that TAD A is required for the activation of *AOX1* and other genes involved in methanol metabolism. To further characterize TAD A and other putative TADs of Mxr1, synthetic transcription factors were constructed by tethering Mxr1ZF to 9aaTADs and expressing them in *Δmxr1* strains. The ability of these constructs to activate the transcription of Mxr1 target genes involved in ethanol and methanol metabolism was examined. We show that Mxr1ZF tethered to three copies of TAD A, but not one copy, activates transcription of Mxr1 target genes. Using a similar approach, we demonstrate that 10 of 20 putative 9aaTADs of Mxr1 are transcriptionally active. Finally, we show that Mxr1 9aaTADs activate *AOX1* transcription only in the presence of General Control Nondepressible 5 (Gcn5), a histone acetyltransferase (HAT), and a component of the SAGA (Spt–Ada–Gcn5 acetyltransferase) complex (27). This suggests that the recruitment of the Gcn5–HAT complex to the promoters of target genes, leading to histone acetylation, is likely the key mechanism by which Mxr1 9aaTADs activate transcription. This study provides new insights into the mechanisms of transcriptional regulation in *K. phaffii*. The findings presented here have potential applications in the design of synthetic transcription factors for precise control of gene expression in industrial yeasts.

## Results

### Regulation of genes of ethanol and methanol metabolism by Mxr1 TAD A

**Figure 1:**
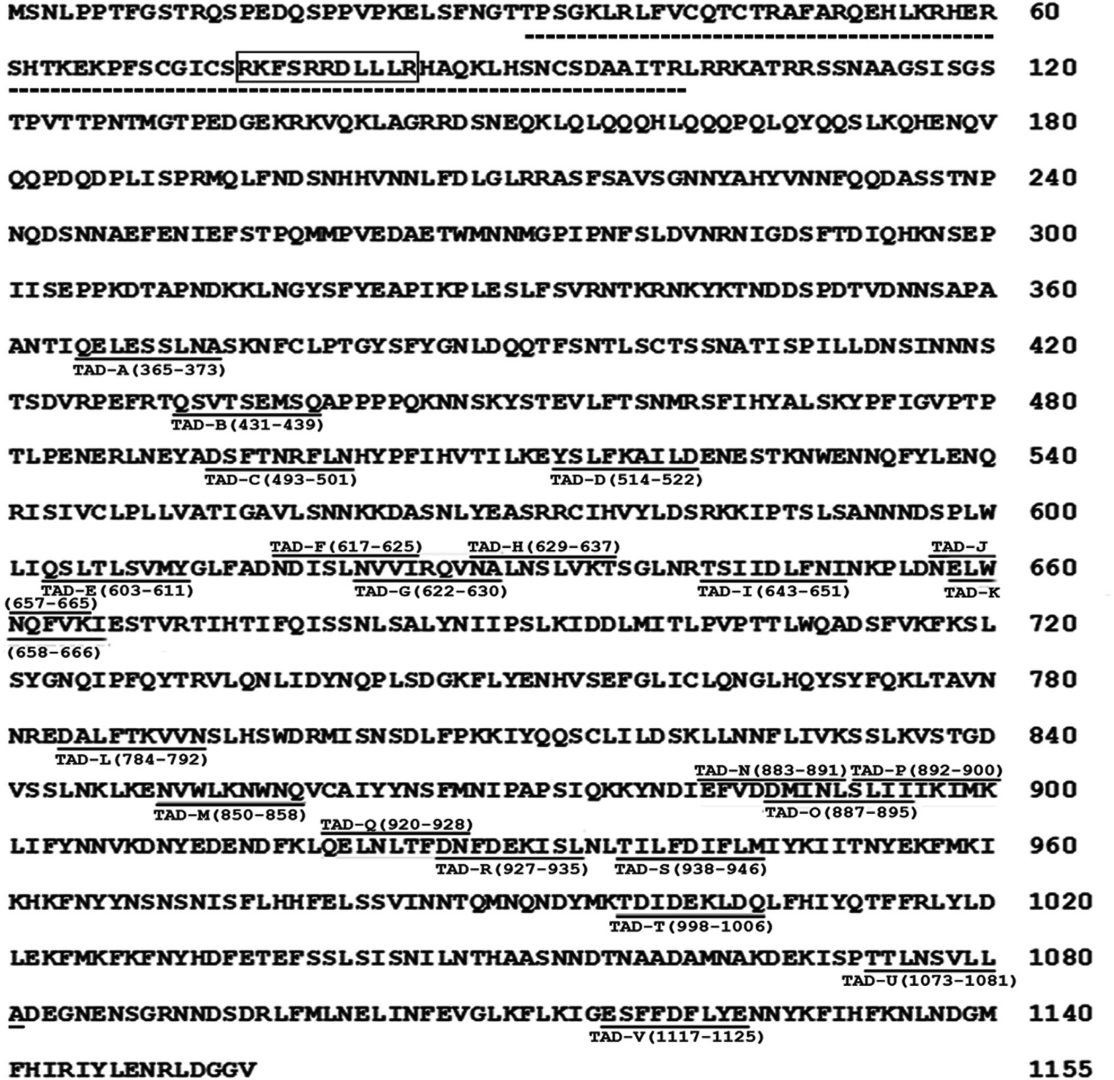
**Amino acid sequence of *Komagataella phaffii* Mxr1 (National Center for Biotechnology Information accession no.: XP_002493922.1).** The DNA-binding domain (DBD) located between amino acids 36 and 101 is highlighted with a *dotted line*. The nuclear localization signal (NLS), spanning amino acids 75 to 85, is enclosed in a *box*. The 9aaTAD (TAD A) and the putative 9aaTADs (labeled B through V) are also marked. 9aaTAD, nine amino acid transactivation domain.

**Figure 2:**
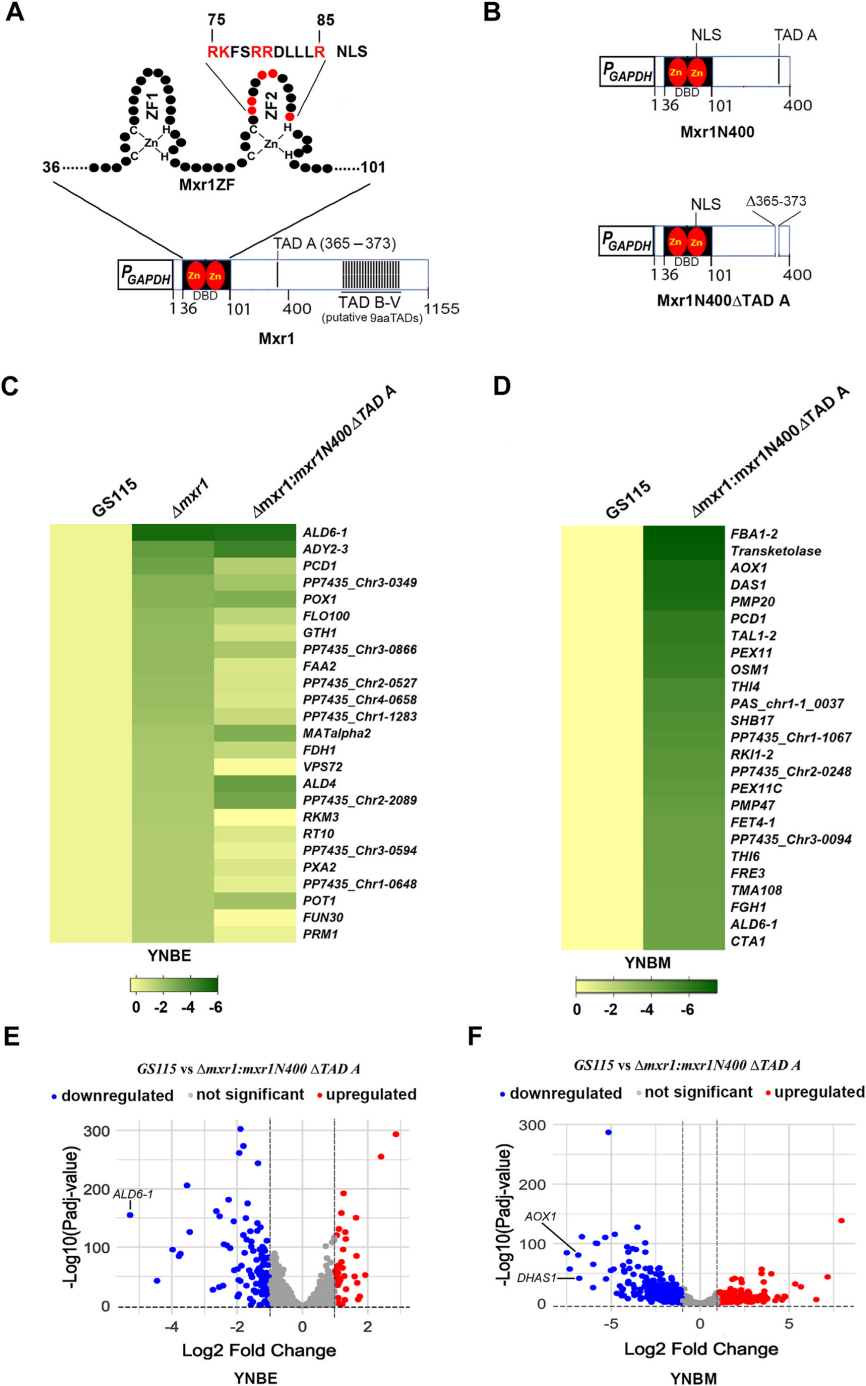
**Key domains of Mxr1 and identification of target genes of TAD A during ethanol and methanol metabolism.***A*, schematic representation of Mxr1. The DNA-binding domain (DBD), which includes two C_2_H_2_ zinc fingers (ZFs), is shown. The nuclear localization signal (NLS) resides within the second ZF, with essential basic amino acid residues (14) highlighted in *red*. The 9aaTAD motif (amino acids 365–373), crucial for ethanol metabolism (14), is designated as TAD A. Additional putative 9aaTAD motifs are also indicated. *B*, schematic diagrams of the *Mxr1N400* and *Mxr1N400ΔTAD A* constructs. *C*, heat map showing genes differentially expressed in *GS115, Δmxr1*, and *Δmxr1:mxr1N400ΔTAD A* cultured in YNBE, plotted as log2 fold changes. The transcriptome dataset for *Δmxr1* was obtained from National Center for Biotechnology Information Gene Expression Omnibus accession number GSE168677. *D*, heat map showing the top 20 downregulated genes in *GS115* and *Δmxr1:mxr1N400ΔTAD A* cultured in YNBM, plotted as log2 fold changes. A full list of differentially expressed genes is provided in Tables S1 and S2. *E*, volcano plot of genes differentially expressed in *GS115* and *Δmxr1:mxr1N400ΔTAD A* cultured in YNBE. The known Mxr1 target gene ALD6-1 is labeled. *F*, volcano plot of genes differentially expressed in *GS115* and *Δmxr1:mxr1N400ΔTAD A* cultured in YNBM. Key Mxr1 target genes AOX1 and DHAS1 are highlighted. 9aaTAD, nine amino acid transactivation domain; AOX1, alcohol oxidase 1; DHAS1, dihydroxyacetone synthase 1; YNBE, yeast nitrogen base and ethanol; YNBM, yeast nitrogen base and methanol.

Our previous study (14) showed that Mxr1N400, consisting of the first 400 N-terminal amino acids of Mxr1, is sufficient to regulate Mxr1 target genes required for ethanol metabolism, such as *ALD6-1*, which encodes aldehyde dehydrogenase, with TAD A being essential for this function. To gain further insights into TAD A function during ethanol metabolism as well as to understand its role during methanol metabolism, *K. phaffii Δmxr1* was transformed with plasmids encoding Mxr1, Mxr1N400, and Mxr1N400ΔTAD (Figs. 1, 2, *A* and *B*) to generate *Δmxr1:mxr1*, *Δmxr1:mxr1N400*, and *Δmxr1:mxr1N400ΔTAD A*, respectively (Table 1). *GS115* and *Δmxr1:mxr1N400ΔTAD A* were cultured in YNBE (0.67% yeast nitrogen base [YNB] and 1% ethanol) for 6 h, RNA was isolated, subjected to RNA-Seq, and the RNA-Seq data were submitted to Bioproject database (https://www.ncbi.nlm.nih.gov/bioproject/1179660). For the analysis, statistical significance was defined as an adjusted *p* value <0.05 and a log2 fold change of ±1 for identifying upregulated and downregulated genes. Analysis of heat map reveals that genes downregulated in Δ*mxr1* remain downregulated in *Δmxr1:mxr1N400ΔTAD A* as well (Fig. 2*C*), indicating that TAD A is required for their transcription during ethanol metabolism. Since the role of Mxr1TAD A during methanol metabolism is not known, RNA-Seq analysis was carried out with RNA isolated from *GS115* and *Δmxr1:mxr1N400ΔTAD A* cultured in YNBM (YNB and 1% methanol) for 6 h, RNA was isolated, and subjected to RNA-Seq. The RNA-Seq data were deposited in the Bioproject database (https://www.ncbi.nlm.nih.gov/bioproject/1179660). The heat map indicates that several known targets of Mxr1, such as *DHAS1* and *AOX1* as well as genes encoding peroxisomal proteins (PMP20, PMP47, and PEX11C), are downregulated in *Δmxr1:mxr1N400ΔTAD A* (Fig. 2*D*), indicating that Mxr1 TAD A is required for their transactivation during methanol metabolism. The overall expression changes in *Δmxr1:mxr1N400ΔTAD A* compared with *GS115*, along with the most prominently upregulated and downregulated genes cultured in YNBE and YNBM, respectively, are visualized in the volcano plots (Fig. 2, *E* and *F*). The list of genes differentially regulated in *Δmxr1:mxr1N400ΔTAD A*, during ethanol and methanol metabolism, is presented in Tables S1 and S2, respectively.

**Table 1:** *K. phaffii* strains used in this study

Yeast strain	Genotype	Reference
*GS115*	*his4*	(7)
*Δmxr1*	*GS115, mxr1:: Zeocin*^*r*^	(12, 13)
*Δgcn5*	*GS115, gcn5:: Zeocin*^*r*^	(30)
*Δmxr1:mxr1*	*Δmxr1, Hyg*^*r*^*::P*_*GAPDH*_*GFP-MXR1*	This study
*Δmxr1:mxr1N400*	*Δmxr1, Bla*^*r*^*::P*_*GAPDH*_*MXR1N400*^*Myc*^	(14)
*Δmxr1:mxr1ΔTAD A*	*Δmxr1, Hyg*^*r*^*::P*_*GAPDH*_*GFP-MXR1ΔTAD A*	This study
*Δmxr1:mxr1N400ΔTAD A*	*Δmxr1, Bla*^*r*^*::P*_*GAPDH*_*MXR1N400ΔTAD A*^*Myc*^	(14)
*Δmxr1:mxr1ZF*	*Δmxr1, Hyg*^*r*^*::P*_*GAPDH*_*mNG-MXR1ZF*	This study
*Δmxr1:mxr1ZF TAD A*^*1x*^	*Δmxr1, Hyg*^*r*^*::P*_*GAPDH*_*mNG-MXR1ZF TAD A*^*1x*^	This study
*Δmxr1:mxr1ZF TAD A*^*3x*^	*Δmxr1, Hyg*^*r*^*::P*_*GAPDH*_*mNG-MXR1ZF TAD A*^*3x*^	This study
*Δmxr1:mxr1ZF TAD C*^*3x*^	*Δmxr1, Hyg*^*r*^*::P*_*GAPDH*_*mNG-MXR1ZF TAD C*^*3x*^	This study
*Δmxr1:mxr1ZF TAD D*^*3x*^	*Δmxr1, Hyg*^*r*^*::P*_*GAPDH*_*mNG-MXR1ZF TAD D*^*3x*^	This study
*Δmxr1:mxr1ZF TAD E*^*3x*^	*Δmxr1, Hyg*^*r*^*::P*_*GAPDH*_*mNG-MXR1ZF TAD E*^*3x*^	This study
*Δmxr1:mxr1ZF TAD F*^*3x*^	*Δmxr1, Hyg*^*r*^*::P*_*GAPDH*_*mNG-MXR1ZF TAD F*^*3x*^	This study
*Δmxr1:mxr1ZF TAD G*^*3x*^	*Δmxr1, Hyg*^*r*^*::P*_*GAPDH*_*mNG-MXR1ZF TAD G*^*3x*^	This study
*Δmxr1:mxr1ZF TAD H*^*3x*^	*Δmxr1, Hyg*^*r*^*::P*_*GAPDH*_*mNG-MXR1ZF TAD H*^*3x*^	This study
*Δmxr1:mxr1ZF-TAD I*^*3x*^	*Δmxr1, Hyg*^*r*^*::P*_*GAPDH*_*mNG-MXR1ZF TAD I*^*3x*^	This study
*Δmxr1:mxr1ZF TAD J*^*3x*^	*Δmxr1, Hyg*^*r*^*::P*_*GAPDH*_*mNG-MXR1ZF TAD J*^*3x*^	This study
*Δmxr1:mxr1ZF TAD K*^*3x*^	*Δmxr1, Hyg*^*r*^*::P*_*GAPDH*_*mNG-MXR1ZF TAD K*^*3x*^	This study
*Δmxr1:mxr1ZF TAD L*^*3x*^	*Δmxr1, Hyg*^*r*^*::P*_*GAPDH*_*mNG-MXR1ZF TAD L*^*3x*^	This study
*Δmxr1:mxr1ZF TAD M*^*3x*^	*Δmxr1, Hyg*^*r*^*::P*_*GAPDH*_*mNG-MXR1ZF TAD M*^*3x*^	This study
*Δmxr1:mxr1ZF TAD N*^*3x*^	*Δmxr1, Hyg*^*r*^*::P*_*GAPDH*_*mNG-MXR1ZF TAD N*^*3x*^	This study
*Δmxr1:mxr1ZF TAD O*^*3x*^	*Δmxr1, Hyg*^*r*^*::P*_*GAPDH*_*mNG-MXR1ZF TAD O*^*3x*^	This study
*Δmxr1:mxr1ZF TAD P*^*3x*^	*Δmxr1, Hyg*^*r*^*::P*_*GAPDH*_*mNG-MXR1ZF TAD P*^*3x*^	This study
*Δmxr1:mxr1ZF TAD Q*^*3x*^	*Δmxr1, Hyg*^*r*^*::P*_*GAPDH*_*mNG-MXR1ZF TAD Q*^*3x*^	This study
*Δmxr1:mxr1ZF TAD R*^*3x*^	*Δmxr1, Hyg*^*r*^*::P*_*GAPDH*_*mNG-MXR1Z TAD R*^*3x*^	This study
*Δmxr1:mxr1ZF TAD S*^*3x*^	*Δmxr1, Hyg*^*r*^*::P*_*GAPDH*_*mNG-MXR1ZF TAD S*^*3x*^	This study
*Δmxr1:mxr1ZF TAD T*^*3x*^	*Δmxr1, Hyg*^*r*^*::P*_*GAPDH*_*mNG-MXR1ZF TAD T*^*3x*^	This study
*Δmxr1:mxr1ZF TAD U*^*3x*^	*Δmxr1, Hyg*^*r*^*::P*_*GAPDH*_*mNG-MXR1ZF TAD U*^*3x*^	This study
*Δmxr1:mxr1ZF TAD V*^*3x*^	*Δmxr1, Hyg*^*r*^*::P*_*GAPDH*_*mNG-MXR1ZF TAD V*^*3x*^	This study
*GS115:P*_*AOX1*_*LacZ*	*GS115, his4*^*+*^*::P*_*AOX1*_*LacZ*	This study
*Δmxr1:mxr1-P*_*AOX1*_*LacZ*	*Δmxr1, Hyg*^*r*^*::P*_*GAPDH*_*GFP-MXR1::his4*^*+*^*::P*_*AOX1*_*LacZ*	This study
*Δmxr1:mxr1N400-P*_*AOX1*_*LacZ*	*Δmxr1, Hyg*^*r*^*::P*_*GAPDH*_*GFP-MXR1N400::his4*^*+*^*::P*_*AOX1*_*LacZ*	This study
*Δmxr1:P*_*AOX1*_*Lac-Z*	*Δmxr1, his4*^*+*^*::P*_*AOX1*_*LacZ*	This study
*Δmxr1:mxr1ZF-TAD A*^*1x*^*-P*_*AOX1*_*LacZ*	*Δmxr1, Hyg*^*r*^*::P*_*GAPDH*_*mNG-MXR1 ZF- TAD A*^*1x*^*:: his4*^*+*^*::P*_*AOX1*_*LacZ*	This study
*Δmxr1:mxr1-ZF-TAD A*^*3x*^*-P*_*AOX1*_*LacZ*	*Δmxr1, Hyg*^*r*^*::P*_*GAPDH*_*mNG-MXR1 ZF-TAD A*^*3x*^*::his4*^*+*^*::P*_*AOX1*_*LacZ*	This study
*Δgcn5:P*_*AOX1*_*LacZ*	*Δgcn5, his4*^*+*^*::P*_*AOX1*_*LacZ*	This study
*Δgcn5:P*_*AOX1*_*LacZ-Mxr1ZF-TAD A*^*3x*^	*Δgcn5, his4*^*+*^*::P*_*AOX1*_*LacZ::Hyg*^*r*^*::P*_*GAPDH*_*MXR1ZF-TAD A*^*3x*^	This study
*Δgcn5:P*_*AOX1*_*LacZ-Mxr1ZF-TAD R*^*3x*^	*Δgcn5, his4*^*+*^*::P*_*AOX1*_*LacZ::Hyg*^*r*^*::P*_*GAPDH*_*MXR1ZF-TAD*^*3x*^	This study
*Δgcn5:P*_*AOX1*_*LacZ-Mxr1ZF-TAD S*^*3x*^	*Δgcn5, his4*^*+*^*::P*_*AOX1*_*LacZ::Hyg*^*r*^*::P*_*GAPDH*_*MXR1ZF-TAD S*^*3x*^	This study
*Δgcn5:P*_*AOX1*_*LacZ-Mxr1ZF-TAD T*^*3x*^	*Δgcn5, his4*^*+*^*::P*_*AOX1*_*LacZ::Hyg*^*r*^*::P*_*GAPDH*_*MXR1ZF-TAD T*^*3x*^	This study

### Transactivation of Mxr1 target genes required for ethanol and methanol metabolism by synthetic transcription factors

**Figure 3:**
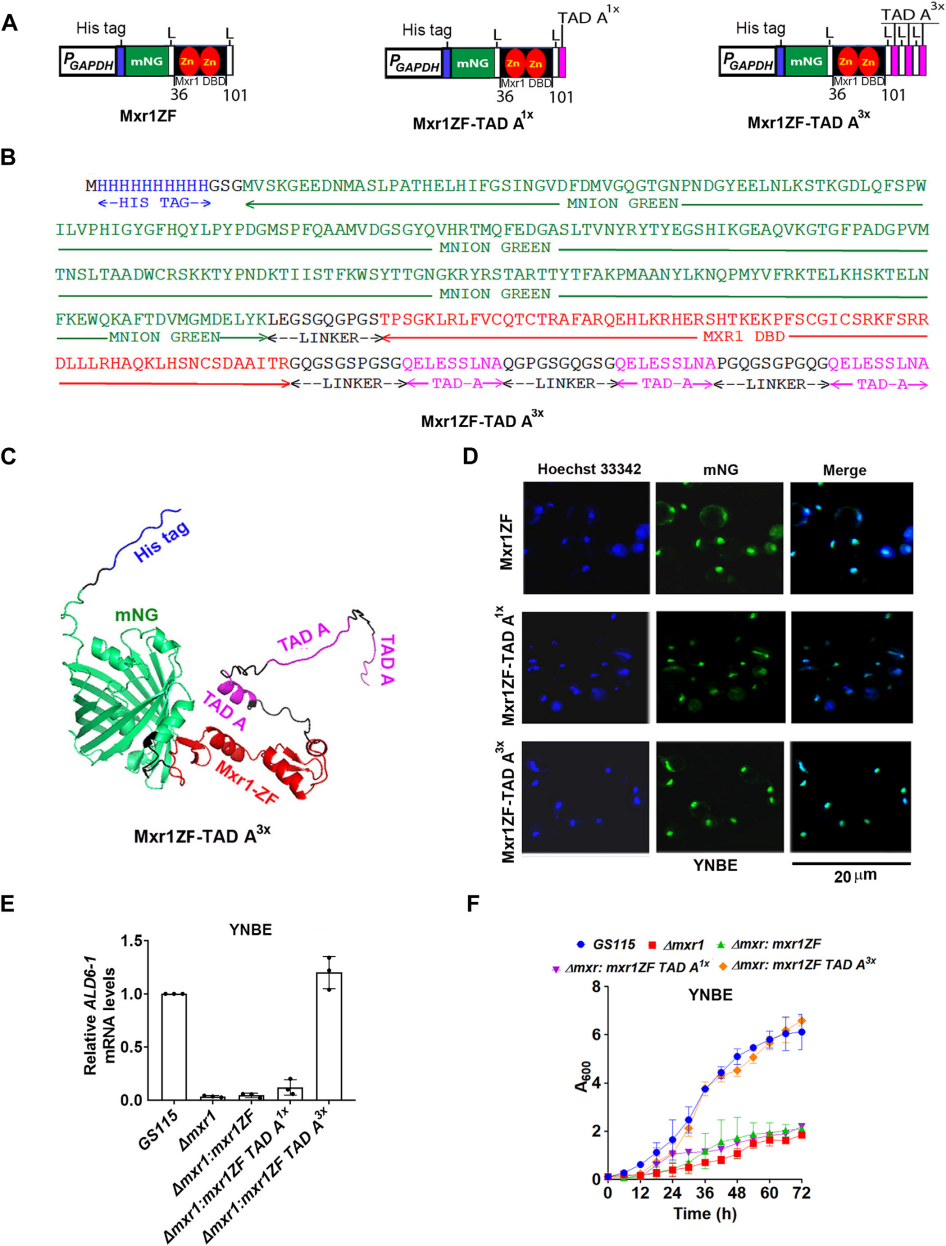
**Design and functional characterization of synthetic Mxr1 transcription factors.***A*, schematic representation of Mxr1ZF, Mxr1ZF tethered to TAD A^1x^ (Mxr1ZF-TAD A^1x^), or TAD A^3x^ (Mxr1ZF-TAD A^3x^). His tag and mNeonGreen (mNG) are included as N-terminal fusions. L, 10 amino acid linker. *B*, amino acid sequence of Mxr1ZF-TAD A^3x^. *C*, predicted structure of Mxr1ZF-TAD A^3x^ by AlphaFold2. *D*, analysis of subcellular localization of Mxr1ZF, Mxr1ZF-TAD A^1x^, and Mxr1ZF-TAD A^3x^ in cells cultured in YNBE for 3 h by fluorescence microscopy. Hoechst 33342 was used to visualize the nucleus. *E*, quantification of *ALD6-1* mRNA levels in different *Komagataella phaffii* strains cultured in YNBE for 6 h by quantitative PCR. Error bars indicate mean ± SD (n = 3). Individual data points indicate the three biological replicates. *F*, analysis of growth of different *K. phaffii* strains cultured in YNBE. Error bars indicate mean ± SD (n = 3). TAD, transactivation domain; YNBE, yeast nitrogen base and ethanol.

To assess whether Mxr1ZF combined with TAD A can function as a synthetic transcription factor, constructs encoding Mxr1ZF alone or fused to either one (Mxr1ZF-TAD A^1x^) or three (Mxr1ZF-TAD A^3x^) copies of TAD A were designed and synthesized, incorporating an N-terminal His tag and mNeonGreen (mNG) for detection (28) (Fig. 3, *A* and *B*). Ten-amino-acid linkers were introduced between mNG and Mxr1ZF, between Mxr1ZF and TAD A, and among the three copies of TAD A in the constructs (Fig. 3, *A* and *B*). *K. phaffii Δmxr1* was transformed with plasmids encoding these proteins to generate *K. phaffii Δmxr1:mxr1ZF*, *Δmxr1:mxr1ZF-TAD A*^*1x*^, and *Δmxr1:mxr1ZF-TAD A*^*3x*^ (Table 1). The 3D structure of Mxr1ZF-TAD A^3x^ was predicted using AlphaFold2 (Figs. 3*C*, S1). To examine whether the NLS present in Mxr1ZF facilitates the nuclear localization of these proteins, mNG fluorescence was examined in live cells cultured in YNBE under a fluorescent microscope. All the three proteins localized to the nucleus (Fig. 3*D*), indicating that NLS in Mxr1ZF is functional. To assess their ability to activate transcription of Mxr1 target genes during ethanol metabolism, *ALD6-1* mRNA levels were quantified by quantitative PCR (qPCR) using RNA isolated from cells cultured in YNBE for 6 h. *ALD6-1* mRNA levels were restored in *Δmxr1* by Mxr1ZF-TAD A^3x^ but not Mxr1ZF-TAD A^1x^ or Mxr1ZF (Fig. 3*E*). Mxr1ZF-TAD A^3x^ reversed the growth defect of *Δmxr1* in YNBE (Fig. 3*F*) indicating that Mxr1ZF-TAD A^3x^ activates transcription of not only *ALD6-1* but also target genes required for growth in ethanol-containing media.

**Figure 4:**
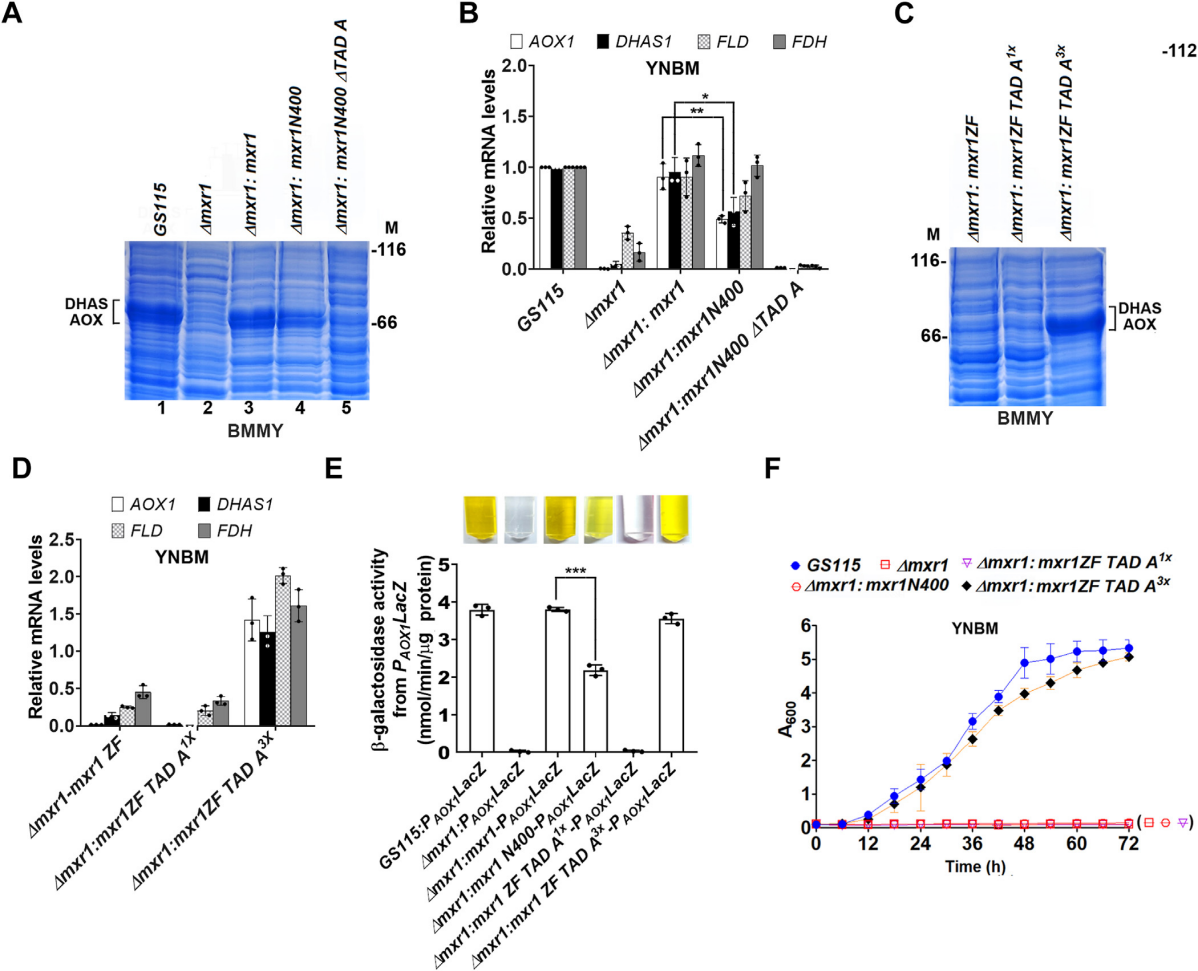
**Analysis of the role of Mxr1 TAD A in the regulation of Mxr1 target genes required for methanol metabolism.***A*, protein profile of *Komagataella phaffii* strains cultured in BMMY medium (1% yeast extract, 2% peptone, 0.1 M potassium phosphate [pH 6.0], 1.34% YNB, 0.0004% biotin, and 1% methanol) as analyzed by SDS-PAGE followed by Coomassie Brilliant *Blue* R staining. M, protein molecular mass markers (kilodalton). The presence of DHAS and AOX in the protein bands was confirmed by mass spectrometry (see supporting information). *B*, quantification of *AOX1*, *DHAS1*, *FLD*, and *FDH* mRNA levels in different *K. phaffii* strains cultured in YNBM for 6 h by quantitative PCR. Error bars indicate mean ± SD (n = 3). *p* Value summary is indicated on the bar of the figure: ∗*p* < 0.05; ∗∗*p* < 0.005; and ∗∗∗*p* < 0.0005 (Student's paired or unpaired *t* test). Individual data points indicate the three biological replicates. *C*, protein profile of *K. phaffii* strains cultured in BMMY medium as analyzed by SDS-PAGE followed by Coomassie Brilliant Blue R staining. M, protein molecular mass markers (kilodalton). *D*, quantification of *AOX1*, *DHAS1*, *FLD*, and *FDH* mRNAs in different *K. phaffii* strains cultured in YNBM for 6 h by quantitative PCR. Error bars indicate mean ± SD (n = 3). Individual data points indicate the three biological replicates. *E*, analysis of *LacZ* activity from *P*_*AOX1*_ in different *K. phaffii* strains cultured in YNBM for 12 h. β-galactosidase activity was quantified. Reaction tubes are shown as *inset*. Error bars indicate mean ± SD (n = 3). ∗∗∗*p* < 0.0005. Individual data points indicate the three biological replicates. *F*, analysis of growth of different *K. phaffii* strains cultured in YNBM. Error bars indicate mean ± SD (n = 3). AOX, alcohol oxidase; DHAS, dihydroxyacetone synthase; TAD, transactivation domain; YNBM, yeast nitrogen base and methanol.

AOX and DHAS are key enzymes in methanol metabolism and are highly expressed during this process, appearing as distinct 74 kDa and 79 kDa bands in SDS-polyacrylamide gels of *GS115* cell lysates (Fig. 4*A*). Mass spectrometry analysis confirmed that these two bands correspond to DHAS and AOX (supporting information). Notably, these two proteins were absent in *Δmxr1* (Fig. 4*A*). Expression of these proteins in *Δmxr1* is restored by Mxr1 as well as Mxr1N400 but not Mxr1N400ΔTAD A (Fig. 4*A*), indicating a key role for TAD A during methanol metabolism as well. It is to be noted that DHAS and AOX levels in *Δmxr1:mxr1N400* are lower than those in Δ*mxr1:mxr1* (compare lanes 3 and 4, Fig. 4*A*), suggesting that transactivation by Mxr1N400 may not be as efficient as that by Mxr1 during methanol metabolism. Analysis of mRNA levels of *AOX1* and *DHAS1*, as well as two other Mxr1 target genes (*FDH* and *FLD*) by qPCR, confirmed downregulation of these mRNAs in *Δmxr1*, which is reversed by the expression of Mxr1 and Mxr1N400 but not Mxr1N400ΔTAD A (Fig. 4*B*). As observed in Fig. 4*A*, Mxr1N400 is not as efficient as Mxr1 in the restoration of *AOX1* and *DHAS1* mRNA levels in *Δmxr1*. These results indicate that TAD A is essential but not sufficient for the complete restoration of expression of *AOX* and *DHAS*. We then examined the ability of the synthetic transcription factors Mxr1ZF-TAD A^1x^ and Mxr1ZF-TAD A^3x^ to regulate the expression of genes of methanol metabolism. As observed during ethanol metabolism, Mxr1ZF-TAD A^3x^ but not Mxr1ZF-TAD A^1x^ or Mxr1ZF activated transcription of Mxr1 target genes during methanol metabolism as evident from protein profile and qPCR analysis (Fig. 4, *C* and *D*). These results were further confirmed by examining *Escherichia coli lacZ* reporter gene expression from *AOX1* promoter (*P*_*AOX1*_). *K. phaffii* strains described previously were transformed with a plasmid expressing *E. coli lacZ* from *P*_*AOX1*_, and β-galactosidase activity was measured in cells cultured in YNBM. Methanol-inducible *LacZ* expression from *P*_*AOX1*_ abrogated in *Δmxr1* was restored by the expression of Mxr1, Mxr1N400, and Mxr1ZF-TAD A^3x^ but not Mxr1ZF-TAD A^1x^ (Fig. 4*E*). β-Galactosidase activity was significantly lower in *Δmxr1:mxr1N400* than in *Δmxr1:mxr1* (Fig. 4*E*), further confirming that Mxr1N400 is not as efficient as Mxr1 in complete restoration of *AOX* promoter activity. Growth defect of *Δmxr1* was restored by Mxr1ZF-TAD A^3x^ but not Mxr1ZF-TAD A^1x^ when cultured in YNBM (Fig. 4*F*). Taken together, these results indicate that TAD A is essential but not sufficient for efficient activation of transcription of Mxr1 target genes required for methanol metabolism.

### Differential regulation of transcription by Mxr1N400ΔTAD A and Mxr1ΔTAD A

**Figure 5:**
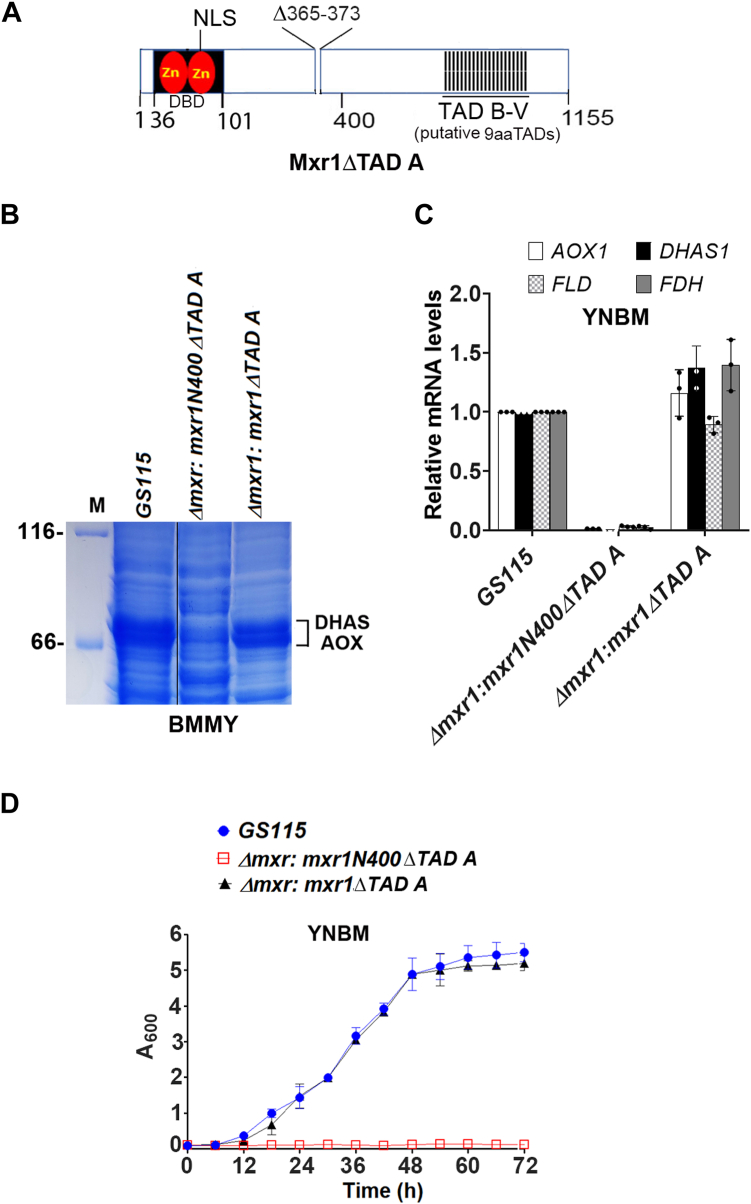
**Comparison of TAD A function in Mxr1N400 *versus* full-length Mxr1.***A*, schematic diagram of Mxr1ΔTAD A. *B*, protein profile of *Komagataella phaffii* strains cultured in BMMY medium as analyzed by SDS-PAGE followed by Coomassie Brilliant Blue R staining. M, protein molecular mass markers (kDa). *C*, quantification of *AOX1*, *DHAS1*, *FLD*, and *FDH* mRNA levels in different *K. phaffii* strains cultured in YNBM for 6 h by quantitative PCR. Error bars indicate mean ± SD (n = 3). Individual data points indicate the three biological replicates. *D*, analysis of growth of *K. phaffii* strains cultured in YNBM. Error bars indicate mean ± SD (n = 3). TAD, transactivation domain; YNBM, yeast nitrogen base and methanol.

The fact that Mxr1N400 only partially restores *AOX1* and *DHAS* mRNA and protein levels in *Δmxr1* and does not correct its growth defect in YNBM suggests that additional putative TADs beyond the first 400 N-terminal amino acids of Mxr1 may be functional during methanol metabolism. To investigate this, we generated *K. phaffii Δmxr1:mxr1ΔTAD A* in which TAD A was deleted in full-length Mxr1 (Fig. 5*A*). Deletion of TAD A in full-length Mxr1 does not result in the downregulation of AOX and DHAS protein (Fig. 5*B*) or *AOX1*, *DHAS1*, *FLD*, and *FDH* mRNA levels (Fig. 5*C*) in *Δmxr1*. Furthermore, Mxr1ΔTAD A but not Mxr1N400ΔTAD A reversed the growth defect of *Δmxr1* cultured in YNBM (Fig. 5*D*). These results indicate that some of the putative TADs located between amino acids 401 and 1155 in Mxr1 may be functional, compensating for the loss of TAD A activity. Furthermore, the inability of Mxr1N400 and Mxr1ZF-TAD A^1x^ to restore the growth of *Δmxr1* suggests that efficient transactivation of Mxr1 target genes, essential for the growth of *K. phaffii* in YNBM, likely requires the coordinated action of multiple TADs within Mxr1.

### Characterization of putative 9aaTADs of Mxr1

**Figure 6:**
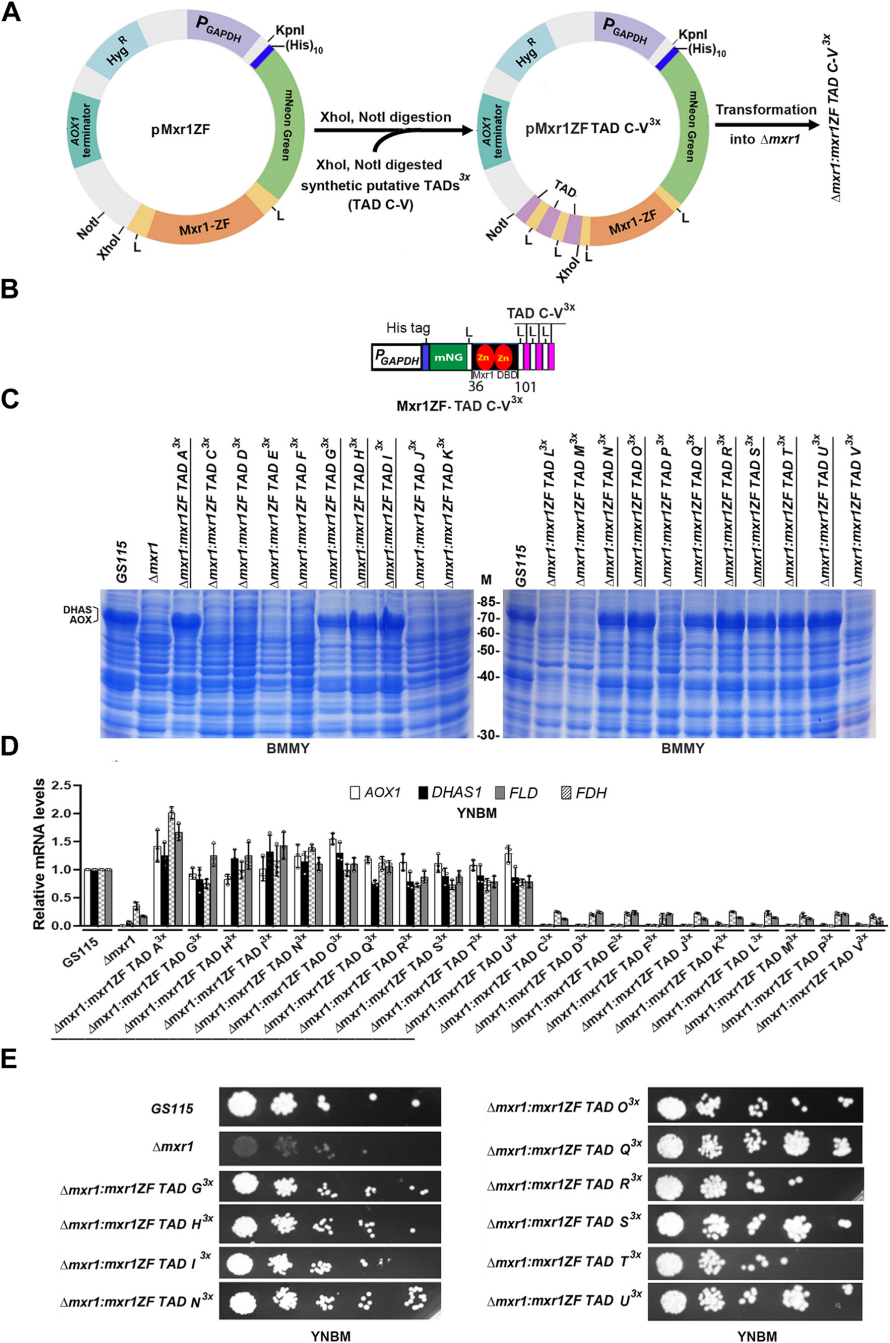
**Identification of Mxr1 9aaTADs that are functional during methanol metabolism.***A*, strategy for the construction of *Komagataella phaffii* strains expressing synthetic proteins harboring Mxr1 DBD and three copies of 20 putative TADs (Mxr1ZF-TAD C-V^3x^). This figure was created using BioRender ((22), https://BioRender.com/s96u589). The nucleotide sequence of synthetic gene fragments is shown in Figure S2. *B*, schematic diagram of Mxr1ZF-TAD C-V^3x^. *C*, protein profile of *K. phaffii* strains cultured in BMMY medium as analyzed by SDS-PAGE followed by Coomassie Brilliant Blue R staining. M, protein molecular mass markers (kilodalton). DHAS and AOX proteins are restored in *K. phaffii* strains expressing functional 9aaTADs (TADs A, G, H, I, N, O, Q, R, S, T, and U). These are underlined. *D*, quantification of *AOX1, DHAS1, FLD*, and *FDH* mRNAs in different *K. phaffii* strains cultured in YNBM for 6 h by qPCR. Error bars indicate mean ± SD (n = 3). Individual data points indicate the three biological replicates. *K. phaffii* strains in which these mRNA levels are comparable to that of *GS115* are underlined. *E*, analysis of growth of *GS115*, *Δmxr1*, and *K. phaffii* strains in which 9aaTADs are functional (TADs G, H, I, N, O, Q, R, S, T, and U). Growth assay was carried out by spotting different dilutions of each *K. phaffii* strain on agar plates containing YNBM. Cells were grown for 72 h at 30 °C. AOX1, alcohol oxidase; 9aaTAD, nine amino acid transactivation domain; DBD, DNA-binding domain; DHAS1, dihydroxyacetone synthase ; YNBM, yeast nitrogen base and methanol.

**Figure 7:**
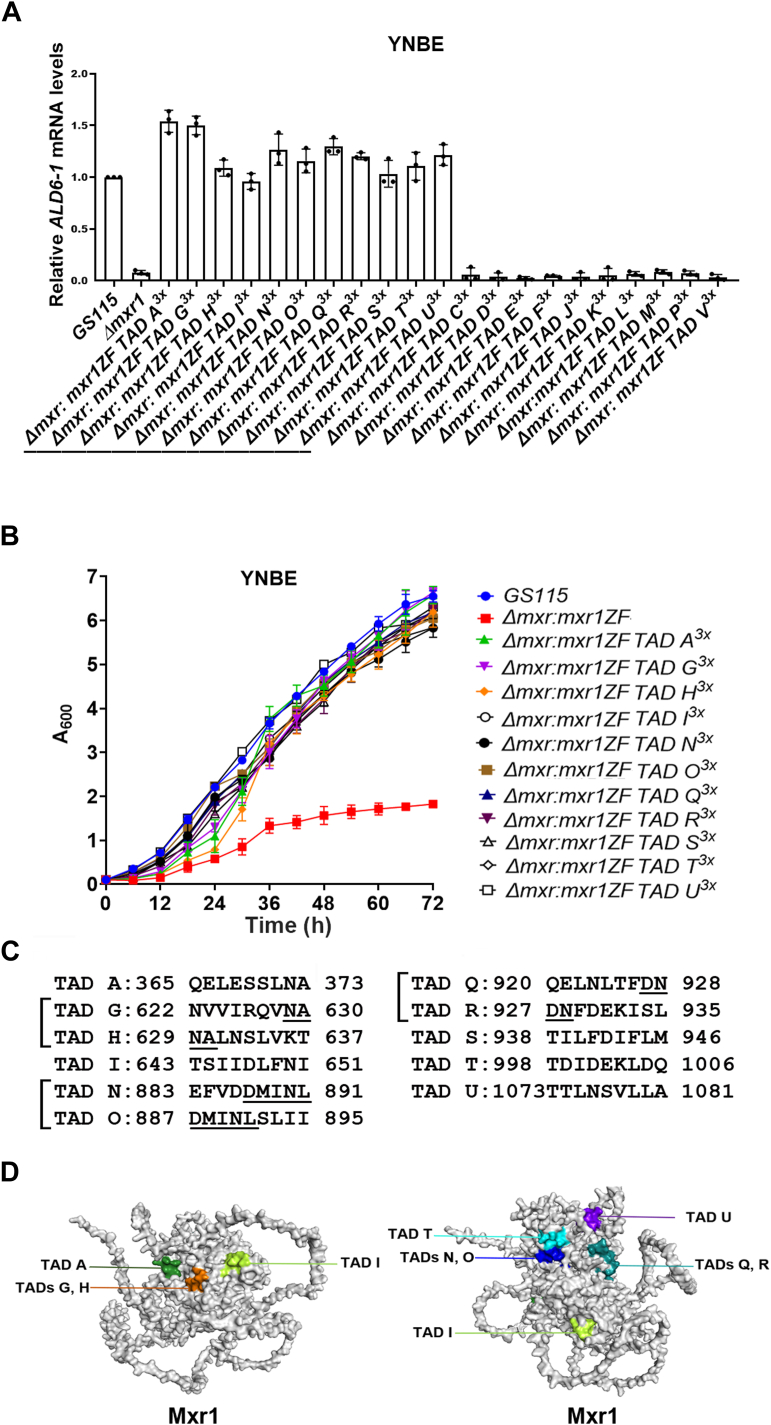
**Identification of Mxr1 9aaTADs that are functional during ethanol metabolism.***A*, quantification of *ALD6-1* mRNA in different *Komagataella phaffii* strains cultured in YNBE for 6 h by quantitative PCR. Error bars indicate mean ± SD (n = 3). Individual data points indicate the three biological replicates. *K. phaffii* strains in which *ALD6-1* mRNA levels are comparable to that of *GS115* are underlined. *B*, comparison of growth of *K. phaffii* strains harboring functional 9aaTADs (TADs A, G, H, I, N, O, Q, R, S, T, and U) with that of *GS115* and *Δmxr1*. Cells were cultured in YNBE up to 72 h. Error bars indicate mean ± SD (n = 3). *C*, amino acid sequence of functional 9aaTADs of Mxr1. TADs G and H, N and O, Q and R contain overlapping amino acid residues (underlined). The position of 10 functional TADs in Mxr1 is shown in Figure S4. *D*, the presence of functional TADs on the surface of Mxr1 as predicted by AlphaFold3. 9aaTAD, nine amino acid transactivation domain; YNBE, yeast nitrogen base and ethanol.

The results thus far suggest that some of the putative 9aaTADs (TADs B–V) of Mxr1 are likely functional during methanol metabolism. Given that a synthetic transcription factor containing three copies of TAD A successfully activated transcription of Mxr1 target genes, a similar strategy was applied to identify functional 9aaTADs among the putative 9aaTADs of Mxr1. Gene fragments encoding three copies of TADs C–V (TAD C–V^3x^) were synthesized (Fig. S2), cloned downstream of Mxr1ZF, and *Δmxr1* was transformed with the resulting plasmids to generate 20 *K. phaffii* strains, *Δmxr1:mxr1ZF TAD C–V*^*3x*^ (Fig. 6, *A* and *B*, Table 1). Due to technical challenges, the synthetic gene encoding TAD B^3x^ (Fig. 1) could not be synthesized and was therefore excluded from this study. Analysis of the subcellular localization of Mxr1ZF TAD C–V^3x^ using mNG fluorescence imaging in live cells shows that all these proteins localize to the nucleus in cells cultured in YNBE (Fig. S3). Analysis of AOX, DHAS protein levels by SDS-PAGE, and *AOX1*, *DHAS1*, *FLD*, and *FDH* mRNA levels by qPCR indicates that TADs G, H, I, N, O, Q, R, S, T, and U are functional during methanol metabolism (Fig. 6, *C* and *D*). These TADs reverse the growth defect of *Δmxr1* as well (Fig. 6*E*), indicating that each of these TADs activate all the Mxr1 target genes required for methanol metabolism. The same TADs are also active during ethanol metabolism, as evident from their ability to activate *ALD6-1* transcription in cells cultured in YNBE (Fig. 7*A*) and restore the growth of *Δmxr1* cultured in YNBE (Fig. 7*B*). Some of these functional 9aaTADs are located in close proximity to one another with overlapping regions (Figs. 7*C*, S4). All the 10 functional TADs except TAD S are predicted to be present on the surface of the protein (Figs. 7*D*, S5).

### Carbon source– and Gcn5-dependent transactivation by Mxr1 9aaTADs

**Figure 8:**
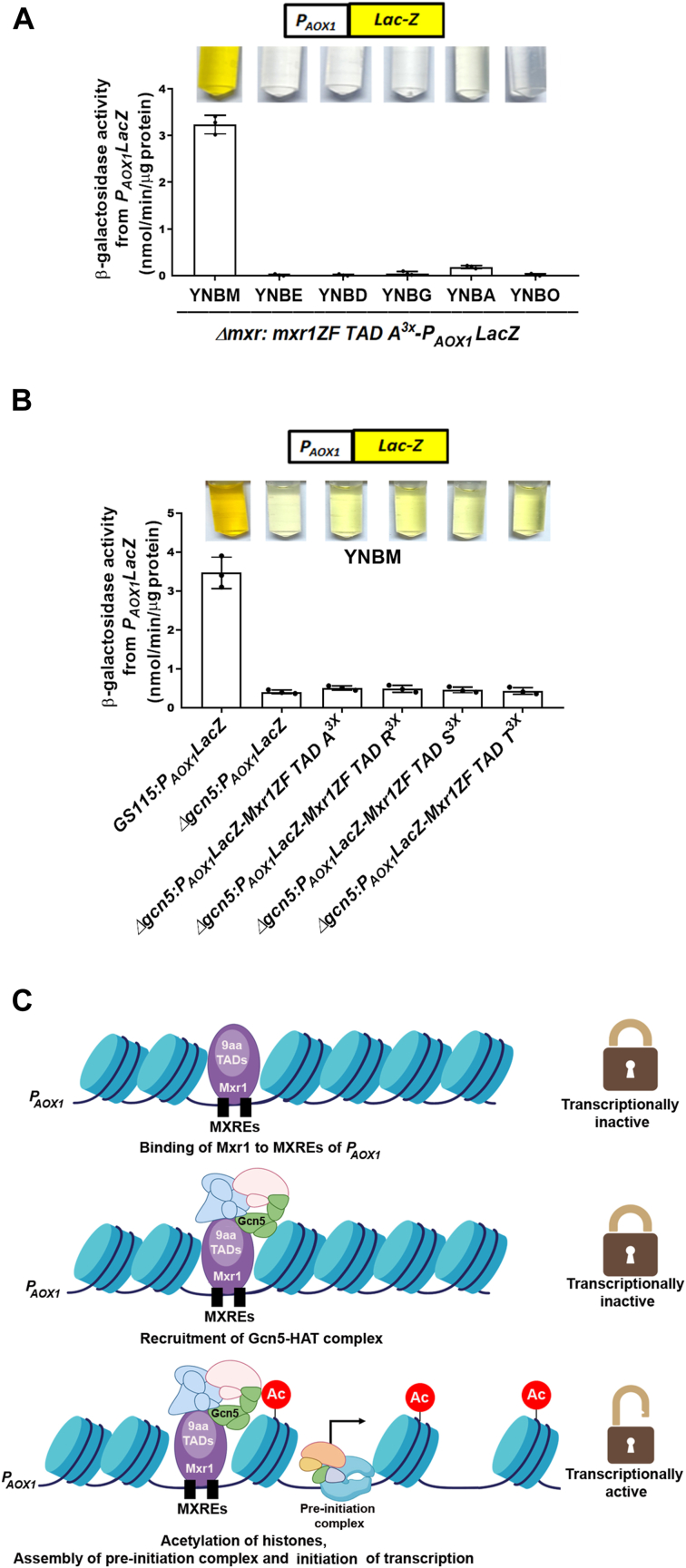
**Analysis of carbon source– and Gcn5-dependent transactivation of *P***_***AOX1***_**by synthetic transcription factors.***A*, methanol-specific transactivation of *P*_*AOX1*_*-LacZ* by the synthetic transcription factor harboring TAD A^3x^. Cells were cultured for 12 h in a medium containing YNB and different sources of carbon, as indicated. β-galactosidase activity was quantified. Reaction tubes are shown as *inset*. Error bars indicate mean ± SD (n = 3). Individual data points indicate the three biological replicates. *B*, transactivation of *P*_*AOX1*_*-LacZ* by *Komagataella phaffii* strains expressing synthetic transcription factors harboring three copies of TADs A, R, S, and T in *GS115* and *Δgcn5*. Cells were cultured in YNBM for 12 h. β-Galactosidase activity was quantified. Reaction tubes are shown as *inset*. Error bars indicate mean ± SD (n = 3). Individual data points indicate the three biological replicates. *C*, a model for Gcn5-dependent, methanol-inducible transactivation of *P*_*AOX1*_ by Mxr1 9aaTADs. This figure was created using BioRender ((22), https://biorender.com/x29r297). 9aaTAD, nine amino acid transactivation domain; YNB, yeast nitrogen base; YNBM, yeast nitrogen base and methanol.

Mxr1 activates transcription of *P*_*AOX1*_ maximally when methanol is the sole source of carbon. To examine whether Mxr1 TAD A also activates *P*_*AOX1*_ with the same specificity as Mxr1, *LacZ* expression from *P*_*AOX1*_ was examined in *Δmxr1:P*_*AOX1*_*LacZ-Mxr1ZF-TAD A*^*3x*^ (Table 1) cultured in media containing methanol, ethanol, glucose, glycerol, acetate, and oleic acid. The results indicate that Mxr1ZF-TAD A^3x^ activated transcription from *P*_*AOX1*_ only when methanol was the sole source of carbon (Fig. 8*A*), suggesting that this 9aaTAD functions in a carbon source–specific manner similar to Mxr1.

**Figure 9:**
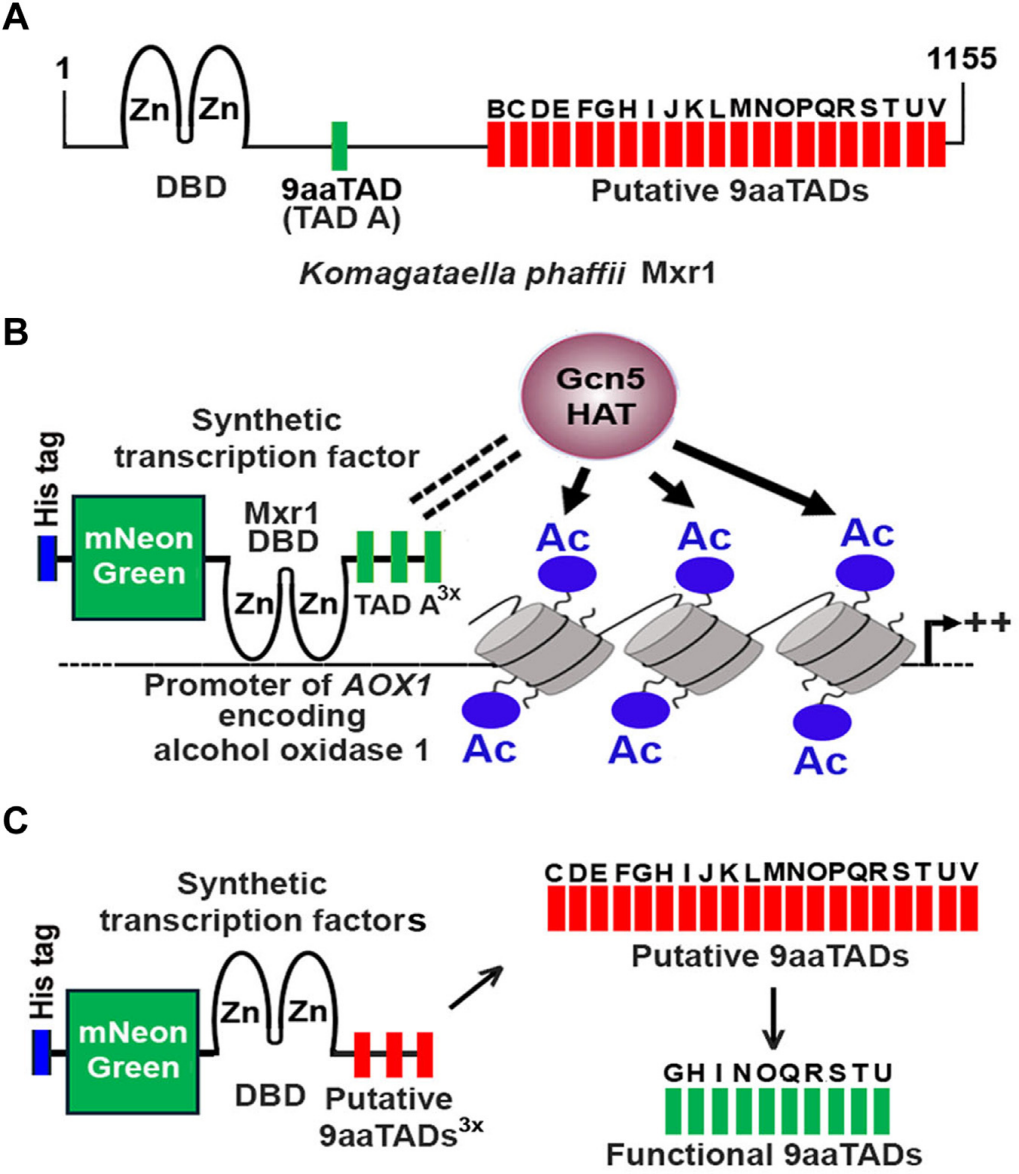
**Schematic representation of Mxr1 and synthetic transcription factors.***A*, Mxr1 comprises a C_2_H_2_ zinc finger DBD, a 9aaTAD, and several, yet-to-be characterized 9aaTADs. *B*, a synthetic transcription factor harboring Mxr1 DBD and three copies of 9aaTAD functions as a Gcn5-dependent transcription factor. *C*, synthetic transcription factors harboring three copies of putative 9aaTADs were designed, and 10 of 20 putative 9aaTADs were found to be functional during ethanol and methanol metabolism. 9aaTAD, nine amino acid transactivation domain; DBD, DNA-binding domain.

In *Saccharomyces cerevisiae*, TADs of transcription factors function by recruiting coactivators such as mediator and SAGA, which form multiprotein complexes with HATs like Gcn5 (29). In *K. phaffii*, methanol-inducible transactivation of *AOX1* is abolished in *Δgcn5* mutant (30), suggesting that the interaction between Mxr1 TADs and Gcn5 may be crucial for the initiation of transcription. To investigate whether Gcn5 is required for the transactivation by the Mxr1 9aaTADs identified in this study, we randomly selected four of the 10 functional 9aaTADs (TADs A, R, S, and T) and assessed their ability to activate *lacZ* expression from *P*_*AOX1*_. β-galactosidase activity was significantly reduced in *Δgcn5* (Fig. 8*B*), indicating that these 9aaTADs likely activate *AOX1* transcription by recruiting the Gcn5 coactivator complex to *P*_*AOX1*_, thereby facilitating histone acetylation (Fig. 8*C*). Key results of this study are summarized in Fig. 9.

## Discussion

This study presents a comprehensive characterization of the TADs in the ZF transcription factor Mxr1 of *K*. *phaffii*. Mxr1 is the recent addition to the list of eukaryotic transcription factors harboring 9aaTADs, which are annotated on the protein UniProt database (https://www.uniprot.org/uniprotkb/?fields=accession%2Creviewed%2Cid%2Cprotein_name%2Cgene_names%2Corganism_name%2Clength&query=9aatad&sort=score&view=table) (24, 31). The 9aaTAD prediction tool (https://www.med.muni.cz/9aaTAD/index.php) facilitates the identification of 9aaTADs in eukaryotic transcription factors by allowing users to apply varying levels of stringency, ranging from less stringent to moderately stringent and most stringent criteria. We have chosen moderate level of stringency to balance sensitivity and specificity; however, not all predictions may be correct. This necessitated functional validation employing the synthetic transcription factor approach. Of the 21 putative 9aaTADs in Mxr1 11 TADs (A, G, H, I, N, O, Q, R, S, T, and U) were functional during both ethanol and methanol metabolism. The prediction tool itself is an evolving resource and is expected to improve in accuracy as more transcription factors are characterized and incorporated into its database. At this stage, it remains challenging to explain why some putative TADs are functional, whereas others are not.

Mxr1N400, which contains only one TAD, is fully functional during ethanol metabolism, whereas the synthetic transcription factor Mxr1ZF-TAD A^1x^, also harboring a single copy of TAD A, is not functional. It is possible that the folding of Mxr1N400 facilitates the interaction of the single copy of TAD A with coactivators such as Gcn5, whereas the synthetic transcription factor Mxr1ZF-TAD A^1x^ does not allow such interactions. This limitation appears to be overcome when multiple copies of TAD A are incorporated, enabling effective interaction and functionality.

Among the 11 functional 9aaTADs, six can be grouped into three pairs based on overlapping amino acid sequences: TAD-G with TAD-H, TAD-N with TAD-O, and TAD-Q with TAD-R (Figs. 7*C*, S4). These pairs may represent longer variants of 9aaTADs, akin to the 14-amino-acid variant observed in NF-κB (32). Multimerization of TADs of transcription factors was shown to improve the cooperativity of transcription factors (33, 34). Our results suggest that tandem repeats of 9aaTADs, as seen in the synthetic factor Mxr1ZF-TAD A^3x^, enhance transcriptional activation, likely through cooperative interactions with transcriptional coactivators. This observation aligns with previous studies on other eukaryotic transcription factors, such as p53 (35), Adr1 (36), Gcn4 (37, 38), CBF-1 (39), and TTF-1 (40), where multimerized TADs exhibited synergistic effects on transcriptional output. The presence of more than one TAD has been shown to help in complex regulation in the case of hypoxia-inducible factor-1α by interaction with two distinct domains of CREB-binding protein (41).

The observation that both Mxr1 and the synthetic transcription factor Mxr1ZF-TAD A^3x^ activate *AOX1* transcription in the presence of methanol, but not ethanol, prompted us to explore the mechanisms of transcriptional repression during ethanol metabolism. In ethanol-metabolizing cells, repression of *AOX1* transcription has been attributed to the phosphorylation of the serine 215 residue, which facilitates interaction with the 14-3-3 protein (42). Substitution of serine 215 with alanine disrupts this interaction, leading to derepression of *AOX1* transcription in ethanol-metabolizing cells (42). However, this regulatory mechanism is not applicable to the synthetic transcription factor, as it consists of only amino acid residues 36 to 101 and lacks serine 215. Alternatively, transcriptional repressors Mig1 and Mig2 are known to bind to the *P*_*AOX1*_ in cells cultured with glycerol, preventing transactivation by Mxr1 (18). It is plausible that Mig1 and Mig2 similarly bind to *P*_*AOX1*_ in cells metabolizing ethanol as well, thereby inhibiting transactivation by both Mxr1 and Mxr1ZF-TAD A^3x^. We note that the results of this study conflict with those of Inoue *et al*. (43), which reported that a mutant Mxr1 protein comprising the 368 N-terminal amino acids, therefore lacking an intact TAD A, was able to activate *AOX1* transcription and restore the growth of *Δmxr1* strain during methanol metabolism. In contrast, our study demonstrates clearly that TAD A is the only functional TAD in Mxr1N400. These discrepancies may arise from differences in the promoters used to express the Mxr1 mutants as well as variations in the methanol concentration in the culture media employed in the two studies.

Analysis of the transactivation function of the synthetic transcription factors in *Δgcn5* shows that *lacZ* expression from *P*_*AOX1*_ is significantly reduced in the absence of Gcn5, highlighting the necessity of Gcn5-mediated histone acetylation. Despite multiple attempts, we were unable to generate a *Δgcn5Δmxr1* double deletion strain, limiting the investigation of 9aaTADs to the presence of Mxr1. Since Mxr1 does not activate transcription in *Δgcn5* (30) (Fig. 8*B*, lane 2), it is unlikely that its presence interferes with the function of synthetic transcription factors. These findings align with the established role of Gcn5 in facilitating transcription by remodeling chromatin and recruiting the transcriptional machinery. It underscores the importance of chromatin context in Mxr1-mediated transcriptional regulation. Prediction of the 3D structure of Mxr1 using AlphaFold3 (43) indicates that all the functional 9aaTADs, except TAD S, are exposed on the surface. This arrangement is likely to enhance their interaction with the coactivator complexes, including the Gcn5–HAT complex. Our findings reveal that synthetic transcription factors containing Mxr1 TAD A^3x^ exhibit methanol-specific activation of the *P*_*AOX1*_ promoter, mimicking the specificity of the full-length Mxr1. This result suggests that TAD activity is modulated by upstream signaling pathways responsive to carbon source availability, adding a layer of regulation to Mxr1 function.

The identification of functional 9aaTADs in Mxr1 opens avenues for engineering synthetic transcription factors with tailored regulatory properties. By leveraging the modular nature of Mxr1 TADs, it may be possible to design synthetic transcription factors optimized for specific metabolic pathways, improving the efficiency and versatility of *K. phaffii* as a recombinant protein expression platform. For instance, synthetic transcription factors combining different 9aaTADs could be used to enhance *P*_*AOX1*_ activity in methanol-free systems or reduce methanol dependence, addressing safety and economic concerns in industrial applications. Furthermore, the insights gained here may facilitate the development of novel expression systems with finely tuned transcriptional responses to environmental or metabolic cues.

This study represents the first detailed exploration of 9aaTADs in a *K. phaffii* transcription factor, enhancing our understanding of their functional diversity and regulatory mechanisms. It focuses on the identification of putative 9aaTADs using the 9aaTAD prediction algorithm and analysis of their functionality using synthetic transcription factors. At this stage, we do not rule out the existence of other TADs besides the 9aaTADs. Future work should focus on exploring the role of Mxr1 TADs in other metabolic pathways, such as acetate, amino acid, and fatty acid metabolism. In addition, investigating the interaction of Mxr1 TADs with other coactivators and chromatin remodelers could provide a deeper understanding of their mechanism of action. These studies have the potential to drive innovation in the field of synthetic biology and industrial biotechnology.

## Experimental procedures

### Growth media and culture conditions

*K. phaffii* cells were maintained on nutrient-rich yeast extract, peptone, and dextrose (YPD) agar plates containing 1% yeast extract, 2% peptone, and 2% dextrose. A single colony was cultured overnight in YPD medium at 30 °C in an orbital shaker set to 180 rpm. Afterward, cells were washed twice with sterile distilled water and transferred to the desired medium containing 0.67% YNB without amino acids and with ammonium sulfate, supplemented with either 1% methanol (YNBM), 1% ethanol (YNBE), 2.0% glucose (YNBD), 2.0% glycerol (YNBG), 2.0% acetate (YNBA), or 2.0% oleic acid (YNBO) as the carbon source. For protein expression, cells initially grown in YPD were transferred to buffered methanol complex medium (BMMY) containing 1% yeast extract, 2% peptone, 0.1 M potassium phosphate (pH 6.0), 1.34% YNB, 0.0004% biotin, and 1% methanol for induction. Yeast extract and peptone were sourced from GIBCO, and YNB from Becton and Dickinson Biosciences. For plasmid isolation, *E. coli DH5α* was used. Transformations in bacteria and yeast were performed using the CaCl₂ method and electroporation (Gene Pulser; Bio-Rad), respectively.

### Generation of *Δmxr1:mxr1* and *Δmxr1:mxr1ΔTAD A*

The gene encoding full-length Mxr1-GFP fusion protein was generated in three PCRs: In the first PCR, *GFP* was amplified from *pGHYBMxr1-250* (14) using forward primer (F1) 5′-CGGGGTACCATGGTGAGCAAGGGCGAG-3′ (KpnI restriction site is underlined) and reverse primer (R1) 5′-AGTTGGGGGTAGATTGCTCATCTTGTACAGCTCG TCCATGCCG-3′ (sequence bearing homology to 5′ end of Mxr1 is underlined) to generate the PCR product P1. In the second PCR, *Mxr1* was amplified from genomic DNA isolated from *K. phaffii GS115* using the forward primer (F2) 5′-CTCGGCATGGACGAGCTGTACAAGATGAGCAATCTACCCCCAACT-3′ (sequence bearing homology to 3′ end of Mxr1 is underlined) and reverse primer (R2) 5′-ATAAGAATGCGGCCGCAGACACCACCATCTAGTCG-3′ (NotI site is underlined) to generate the PCR product P2. The overlap extension PCR was carried out with P1 and P2 as templates and F1 and R2 as primers to generate the PCR product P3 encoding GFP-Mxr1fusion protein. P3 was digested with KpnI and NotI and cloned into *pGHYB* (Addgene, plasmid #87447) to generate *pGHYB-GFP-Mxr1*, which was transformed into *E. coli DH5α*. The recombinant plasmid was isolated from hygromycin-resistant colonies and linearized with AvrII*. K. phaffii Δmxr1* was transformed with the linearized plasmid by electroporation to generate *K. phaffii Δmxr1:mxr1*-*GFP*. GFP-Mxr1–expressing transformants were screened by fluorescence microscopy.

The gene encoding GFP-Mxr1ΔTAD A fusion protein was generated in three PCRs: In the first PCR, the gene encoding amino acids 40 to 364 of Mxr1 was amplified from *pGHYB-GFP-Mxr1*, using the forward primer (F1) 5′-TTTGTCTGTCAGACATGTACTCGAGCATTTGCTCGTCAGGAACAC-3′ (XhoI site is underlined) and reverse primer (R1) 5′-GGCAAGCAAAAATTCTTGGAAATGGTAT TAGCAGCCGGTG-3′ (sequence bearing homology to the region downstream of codon encoding amino acid 373 is underlined) to generate the product, P1. In the second PCR, the Mxr1 region beyond the codon encoding amino acid 373 was amplified from plasmid *PGHYB-GFP-Mxr1* using forward primer (F2) 5′-CACCGGCTGCTAATACCATTTCCAAGAATTTTTGCTTGCC-3′ (sequence bearing homology to the region upstream of codon encoding amino acid 365 is underlined) and reverse primer (R2) 5′-TCTAGAAAGCTGGCGGCCCTCGAGCTAGCAGACACCACCATCTAG-3′ (XhoI site is underlined) to generate the product P2. The final PCR was carried out with P1 and P2 as templates and F1 and R2 as primers to generate the PCR product P3 encoding GFP-Mxr1ΔTAD A fusion protein. P3 was digested with XhoI and cloned into *PGHYB-GFP-Mxr1* using NEBuilder Hifi DNA Assembly to generate *pGHYB-GFP-Mxr1ΔTAD A*, which transformed into *E. coli DH5α*. The plasmid was purified from hygromycin-resistant colonies and linearized with AvrII*. K. phaffii Δmxr1* was transformed with the linearized plasmid by electroporation to generate *K. phaffii Δmxr1:mxr1* expressing GFP-Mxr1ΔTAD A, which was visualized by fluorescence microscopy.

### Generation of *Δmxr1:mxr1ZF*, *Δmxr1:mxr1ZF-TAD A*^*1x*^, and *Δmxr1:mxr1ZF-TAD A*^*3x*^

To generate *K. phaffii* Δ*mxr1*:*mxr1ZF-TAD A*^*3x*^, a synthetic gene encoding mNG fused to *Mxr1ZF*, and three copies of TAD A were synthesized by Twist Bioscience. The gene coded for an N-terminal His tag followed by mNG, Mxr1ZF and three copies of TAD A, each separated by 3 to 10 amino acid linkers. KpnI and NotI restriction sites were introduced at the 5′ and 3′ ends, respectively. The gene was cloned into the *pGHYB* (Addgene plasmid #87447) to generate the plasmid p*Mxr1ZF-TAD A*^*3x*^. This plasmid was transformed into *E. coli DH5α*, and recombinant clones were identified by PCR amplification of the plasmid using gene-specific primers as well as KpnI and NotI digestion. Recombinant plasmid was linearized with *AvrII* and transformed into *K. phaffii Δmxr1* by electroporation to generate *Δmxr1:mxr1ZF-TAD A*^*3x*^. Positive colonies were screened for mNG expression using fluorescence microscopy.

The gene encoding Mxr1ZF-TAD A^1x^ was generated by PCR amplification of *pMxr1ZF-TAD A*^*3x*^ as template and the primer pair 0.5′-GTGGCCCAGCCGGCCGTCTCGGATCGGTACCGCCATGGCTCACCACCACCACCACCACCACCAC-3′ and 5′-CTGGCGGCCGC**TCA**GGCATTCAAACTAGACTCCAACTC TTGACCGGATCCGGGAGAACCTGACCCTTGACCTCTAGTGATAGCTGCGTCAGAACA-3’. Stop codon is shown in boldface, and NotI site is underlined. The PCR product was digested with KpnI and NotI and cloned into the *pGHYB* vector (Addgene plasmid #87447) to generate the plasmid *pMxr1ZF-TAD A*^*3x*^. This plasmid was transformed into *E. coli DH5α*, and the resulting colonies were verified by PCR amplification using gene-specific primers as well as KpnI and NotI digestion. Recombinant plasmid was linearized with AvrII and transformed into *K. phaffii Δmxr1* by electroporation to generate *Δmxr1:mxr1ZF-TAD A*^*1x*^. Recombinant clones expressing Mxr1ZF-TAD A^1x^ were scored for mNG expression in a fluorescence microscope.

The gene encoding Mxr1ZF was generated in a PCR using *pMxr1ZF-TAD A*^*3x*^ as the template and the primer pair 5′-GTGGCCCAGCCGGCCGTCTCGGATCGGT ACCGCCATGGCTCACCACCACCACCACCACCACCAC-3′ and 5′-GAGATGAGTTTTTGTTCTAGAAAGCTGGCGGCCGC**TCA**CTCGAGACCGGATCCGGGAGAACCTGACCC-3’. XhoI and NotI restriction sites are underlined, and stop codon is shown in boldface). The PCR product was digested with KpnI and NotI and cloned into the *pGHYB* vector (Addgene plasmid #87447) to generate the *pMxr1ZF*. The recombinant plasmid was linearized with AvrII and transformed into *K. phaffii Δmxr1* by electroporation to generate *K. phaffii Δmxr1:mxr1ZF*. Positive colonies were screened for mNG expression using fluorescence microscopy.

### Generation of *K. phaffii Δmxr1*:*mxr1ZF-TAD C-V*^*3x*^

Twenty synthetic gene fragments (*TAD C*^*3x*^ to *TAD V*^*3x*^) of 194 bp size encoding three tandem repeats of putative 9aaTADs C to V were synthesized by Genscript (Fig. S2). XhoI and NotI restriction sites were introduced at the 5′ and 3′ ends, respectively. These synthetic gene fragments were cloned at XhoI and NotI sites of *pMxr1ZF*, downstream of Mxr1ZF. Recombinant plasmids were linearized with AvrII. *K. phaffii Δmxr1* strain was transformed with 20 linearized plasmids to generate 20 *K. phaffii* strains, *Δmxr1*:*mxr1ZF-TAD C-V*^*3x*^. Expression of mNG-fusion proteins and their nuclear localization was confirmed by fluorescence microscopy.

### Generation of *GS115:P*_*AOX1*_*LacZ*, *Δmxr1:P*_*AOX1*_*Lac-Z*, *Δmxr1:mxr1-P*_*AOX1*_*LacZ*, *Δmxr1:mxr1N400-P*_*AOX1*_*LacZ*, *Δmxr1:mxr1ZF TAD A*^*1x*^*-P*_*AOX1*_*LacZ*, *Δmxr1:mxr1ZF TAD A*^*3x*^*-PAOX1LacZ*, and *Δgcn5:PAOX1LacZ*

*pP*_*AOX1*_*-LacZ* was generated by carrying out three PCRs. In the first PCR, *P*_*AOX1*_ was amplified from *K. phaffii GS115* genomic DNA using forward primer (F1) 5′-ACTAGTCTCGAGGATCTAACATCCAAAGACG-3′ (XhoI site underlined) and reverse primer (R1) 5′-GGCGTAATCATGGTCATCGTTTCGAATAATTAGTT-3′ (sequence bearing homology to *E. coli LacZ* is underlined) to generate the PCR product P1. In the second PCR, the *LacZ* was amplified from pUC19 using forward primer (F2) 5′-AACTAATTATTCGAAACGATGACCATGATTACGCC-3′ (sequence homologous to *P*_*AOX1*_ is underlined) and reverse primer (R2) 5′-CAAGACCGGTT**CTA**TGCGGCATCAGAGCAG-3′ (stop codon is shown in bold face, and AgeI restriction site is underlined) to generate the PCR product P2. The final PCR was carried out with P1 and P2 as templates and F1 and R2 as primers to generate the PCR product P3 harboring *P*_*AOX1*_*-LacZ*. P3 was digested with XhoI and AgeI and cloned into *pIB3* (Addgene plasmid #25452) to generate *pIB3-P*_*AOX1*_*-LacZ*, which transformed into *E. coli DH5α*. The recombinant plasmid was linearized with AvrII. *GS115*, *Δmxr1*, *Δmxr1:mxr1*, *Δmxr1:mxr1N400*, *Δmxr1:mxr1ZF-TAD A*^*1x*^, *Δmxr1:mxr1ZF-TAD A*^*3x*^, and *Δgcn5* were transformed with the linearized plasmid by electroporation to generate *GS115:P*_*AOX1*_*LacZ*, *Δmxr1:P*_*AOX1*_*Lac-Z*, *Δmxr1:mxr1-P*_*AOX1*_*LacZ*, *Δmxr1:mxr1N400-P*_*AOX1*_*LacZ*, *Δmxr1:mxr1ZF-TAD A*^*1x*^*-P*_*AOX1*_*LacZ*, *Δmxr1:mxr1ZF-TAD A*^*3x*^*-P*_*AOX1*_*LacZ*, and *Δgcn5:P*_*AOX1*_*LacZ*, respectively.

### Generation of *Δgcn5-P*_*AOX1*_*LacZ-Mxr1ZF-TAD A*^*3x*^, *Δgcn5-P*_*AOX1*_*LacZ-Mxr1ZF-TAD R*^*3x*^, *Δgcn5-P*_*AOX1*_*LacZ-Mxr1ZF-TAD S*^*3x*^, and *Δgcn5-P*_*AOX1*_*LacZ-Mxr1ZF-TAD T*^*3x*^

*K. phaffii Δgcn5* was first transformed with *pIB3-P*_*AOX1*_*-Lac-Z*, linearized with SalI to generate Δ*gcn5*-*P*_*AOX1*_-*LacZ*, which was retransformed with AvrII linearized *pMxr1ZFTAD A*^*3x*^, *pMxr1ZFTAD R*^*3x*^, *pMxr1ZF-TAD S*^*3x*^, and *pMxr1ZF-TAD T*^*3x*^ to generate *Δgcn5-P*_*AOX1*_*LacZ-Mxr1ZF-TAD A*^*3x*^, *Δgcn5-P*_*AOX1*_*LacZ-Mxr1ZF-TAD R*^*3x*^, *Δgcn5-P*_*AOX1*_*LacZ-Mxr1ZF-TAD S*^*3x*^, and *Δgcn5-P*_*AOX1*_*LacZ-Mxr1ZF-TAD T*^*3x*^, respectively. Recombinant clones expressing mNG fusion proteins were identified by fluorescent microscopy.

### Growth kinetics

A single colony from the culture plate was transferred into 5 ml of YPD liquid medium and incubated at 30 °C with shaking at 180 rpm overnight or until the late logarithmic phase. The cells were then pelleted by centrifugation, washed three times with sterilized Milli-Q water under sterile conditions, and resuspended in fresh water. The cell suspensions were inoculated into YNBE or YNBM at an initial absorbance of 0.1/ml at 600 nm and cultured at 30 °C in an orbital shaker set to 180 rpm. Absorbance at 600 nm was measured periodically until the cultures reached the stationary phase.

### Spot assay

Cells were cultured overnight at 30 °C with shaking at 180 rpm in YPD medium. The cells were then pelleted by centrifugation and washed three times with sterile distilled water under aseptic conditions. The washed cells were resuspended to prepare a suspension with an absorbance of 0.06 to 0.1 at 600 nm (designated as dilution 1). This suspension was serially diluted five times using a 10-fold dilution series. From each dilution, 2 μl was spotted onto YNBM-agar plates in order of decreasing concentration. The plates were incubated at 30 °C for 72 h.

### Live cell imaging

Cells were cultured in YPD overnight and then transferred to the respective minimal media as described in the *Culture Media* section. After 6 h of induction, 500 μl of cells were pelleted down by centrifuging at 5000 RPM for 2 min and then washed with 1X PBS (137 mM NaCl, 2.7 mM KCl, 4.3 mM Na_2_HPO_4,_ and 1.47 mM KH_2_PO_4_). Adjusted to a final pH of 7.4 twice and then resuspended into 500 μl of 1X PBS. Ten microliters of Hoechst 33342 was then added to the resuspended cells and incubated at 30 °C for 10 min at 180 RPM. The cells were rewashed with 1X PBS twice postincubation. The Olympus IX83 widefield microscope equipped with a CoolLED PE-4000 LED light source and Prime-BSI ScMOS camera was used for acquiring images. All the images were acquired in a 100x oil-immersion objective, 1 μm step-size, and three z-slices. The raw images were processed for 3D deconvolution using Olympus CellSens Dimension (3.1) software. Maximum-intensity projection images were used for representation.

### RNA-Seq and isolation

Total RNA was extracted from four biological replicates (n = 4) of *GS115* and *Δmxr1:mxr1N400ΔTAD A* strains cultured for 6 h in YNBE or YNBM using the Qiagen RNeasy kit. RNA-Seq was conducted with Illumina HiSeq, and data quality was assessed using FastQC and MultiQC, showing high-quality results (Q20 >95%). Fastp removed adapters and low-quality bases, and Bowtie2 aligned the reads to the *GS115* genome. Gene expression levels were quantified with feature counts, and biological replicate similarity was confirmed through Spearman correlation and principal component analysis. Differential expression analysis was performed with DESeq2, filtering for genes with at least five reads, variance-stabilized normalization, and differential enrichment analysis. Significant genes (absolute log2 fold change ≥1, adjusted *p* ≤ 0.05) were visualized in the volcano and heat maps. Functional annotations were obtained using BLASTp and UniProtKB, with enriched Gene Ontology categories retrieved from Amigo. Data are available in the National Center for Biotechnology Information under accession number PRJNA1179660.

### Real-time qPCR

*K. phaffii* cells were cultured in YPD overnight, pelleted down, and washed thrice with sterile distilled water. Cells were then transferred to induction medium (YNBM or YNBE), cultured for 6 h, and total RNA was extracted using an RNA isolation kit (Promega; catalog no.: Z3100) according to the manufacturer's protocol. RNA was quantified by measuring absorbance at 260 nm on a NanoDrop 2000 spectrophotometer. For complementary DNA synthesis, 1 μg of DNase-treated RNA was used. qPCR was conducted with the iQ SYBR Green super mix on a StepOnePlus Real-Time PCR system (Thermo Fisher Scientific). The ΔΔCt method was used to calculate relative mRNA expression levels, where the ΔCt for each sample (gene Ct relative to 18S rRNA Ct) was normalized to the control sample. The final graph contains data from three biological replicates of each strain. The following primer pairs were used: *AOX1*: 5′-AACTTGTCTGCTGGTTCTT-3′ and 5′-CCTTGTCATCCTCCTCAT-3′; *DHAS1*: 5′-CTATTGTTGGTGATGCTTG-3′ and 5′-CCTGGTTGTTGTCGTAGA-3′; *FLD*: 5′-ATGT CTACCGAAGGTCAAACAT-3′ and 5′-GGAGCTTCGTCTTGGACTTTAACC-3′; *FDH*: 5′-ATGAAATCGTTCTCGTTTTTAC-3′ and 5′-ATGGTCTTACCTTCGATATCGTA AG-3′; and *ALD6*-*1*: 5′-GTTCCAGAGTCTACATTCAAG-3′ and 5′-TTACACGAGCACCT TCAT-3′.

### Yeast cell lysis and analysis of protein profile by SDS-PAGE

Yeast cells were cultured in BMMY medium at 30 °C for 12 to 16 h. Cells equivalent to an absorbance of 5 at 600 nm were collected by centrifugation at 5000 rpm for 5 min, then washed with 1 ml of 20% (w/v) trichloroacetic acid. The cell pellet was resuspended in 200 μl of 20% trichloroacetic acid, combined with an equal volume of 0.5 mm glass beads, and subjected to mechanical disruption *via* bead-beating (1 min of lysis followed by 1 min on ice) for four cycles. The lysate was centrifuged at 14,000 rpm for 15 min at 4 °C to pellet the precipitated proteins. The supernatant was removed, and the protein pellet was resuspended in 1X SDS-PAGE loading dye. Proteins were resolved on a 10 to 12% SDS-PAGE gel and stained with Coomassie Brilliant Blue R-250 for visualization. Stained gel was scanned using HP Scanjet G3110 in 600 dpi resolution.

### **β**-galactosidase assay

β-galactosidase activity was measured using ortho-nitrophenyl-d-galactopyranoside as the substrate. Cells were cultured in YNBM for 12 to 16 h. A 50 μg sample of whole cell protein lysate was diluted with 1 ml of Z-buffer (60 mM Na₂HPO₄, 40 mM NaH₂PO₄, 10 mM KCl, 1 mM MgCl₂, pH 7.0) and incubated for 5 min at 30 °C. Then, 0.2 ml of a 4 mg/ml ortho-nitrophenyl-d-galactopyranoside stock solution (diluted in 100 mM sodium phosphate buffer, pH 7.0) was added, and the reaction was performed at 30 °C for 5 to 10 min. Then 0.25 ml of 2M Na₂CO₃ was added to stop the reaction. The release of o-nitrophenol was quantified by measuring absorbance at 420 nm. Enzyme activity was defined as the amount (nanometer) of o-nitrophenol released per minute per microgram of protein lysate under these conditions.

### 3D structure prediction of proteins

3D structures of Mxr1ZF-TAD A^3x^ and all 20 synthetic proteins were predicted by inputting the respective sequences in AlphaFold2 (44). 3D structure prediction of full-length Mxr1 was obtained using AlphaFold3 (45). The predicted structures were visualized in PyMOL (2.6) software.

### Statistical analysis

Statistical analyses were conducted using one-way ANOVA, followed by Tukey’s multiple comparison test in GraphPad Prism 5 (GraphPad Software, Inc). Data are presented as mean ± SD, with significance levels indicated as follows: ∗for *p* < 0.05, ∗∗for *p* < 0.005, ∗∗∗for *p* < 0.0005, and “ns” for nonsignificant differences.

## Data availability

The RNA-Seq data were deposited in the Bioproject database under accession number PRJNA1179660 (https://www.ncbi.nlm.nih.gov/bioproject/1179660).

## Supporting information

This article contains supporting information.

## Conflict of interest

The authors declare that they have no conflicts of interest with the contents of this article.
