# Supplementary material for: Synthetic transcription factors establish the function of nine amino acid transactivation domains of Komagataella phaffii Mxr1
Source: J Biol Chem. 2025 Jan 22;301(3):108211. doi: 10.1016/j.jbc.2025.108211 (PMC11872449; doi:10.1016/j.jbc.2025.108211)
Supplement: Supporting information data [file mmc1.pdf]

MATRIX SCIENCE Mascot Search Results

User	:	Prachi
Email	:	msfacilityiisc@gmail.com
Search title	:	
MS data file	:	D:\Data\lcms\2024\Prachi_Priya\PP_PNR_Prachi-(1).mgf
Database	:	Prachi 20240905 (2 sequences; 1373 residues)
Timestamp	:	5 Sep 2024 at 06:01:44 GMT
Warning	:	No taxonomy indexes found in selected databases, taxonomy 'Saccharomyces Cerevisiae (baker's yeast)' ignored. Sear
Enzyme	:	Trypsin
Fixed modifications	:	Carbamidomethyl (C)
Variable modifications	:	Oxidation (M)
Mass values	:	Monoisotopic
Protein Mass	:	Unrestricted
Peptide Mass Tolerance	:	± 10 ppm
Fragment Mass Tolerance	:	± 0.8 Da
Max Missed Cleavages	:	1
Instrument type	:	Default
Number of queries	:	33272
Protein hits	:	Alcohol oxidase [Komagataella phaffii GS115] Alcohol oxidase [Komagataella phaffii GS115]<br>Dihydroxyacetone synthase variant 1 [Komagataella phaffii CBS 7435] Dihydroxyacetone synthase variant 1 [Komagatae

Select Summary Report

Format As: Export Search Results — Help

Significance threshold p< 0.05 — Max. number of hits AUTO

Standard scoring ○ MudPIT scoring ◉ Ions score or expect cut-off 0 — Show sub-sets 0

Show pop-ups ◉ Suppress pop-ups ○ — Require bold red ☐

Preferred taxonomy: All entries

Re-Search — ◉ All queries ○ Unassigned ○ Below homology threshold ○ Below identity threshold

1. Alcohol oxidase [Komagataella phaffii GS115] **Mass:** 74812 **Score:** 1273 **Matches:** 297(118) **Sequences:** 61(38) **emPAI:** 13.62

Alcohol oxidase [Komagataella phaffii GS115]

Query	Observed	Mr(expt)	Mr(calc)	ppm	Miss	Score	Expect	Rank	Unique	Peptide
139	355.6896	709.3647	709.3646	0.04	0	13	0.053	1	U	K.LGTYEK.T
217	360.1936	718.3727	718.3722	0.74	1	7	0.19	1	U	K.SRETAR.R 208 209 210 212 213 214 216 218 219
514	383.7083	765.4021	765.4021	0.04	0	14	0.039	1	U	R.SNVYGVK.G 513 515
858	399.1933	796.3720	796.3715	0.66	0	11	0.085	1	U	K.TETYQR.A 856 857 859 860
907	401.2264	800.4383	800.4392	-1.10	0	25	0.0032	1	U	K.IIVEDGR.A 909 911 912
1112	410.7138	819.4130	819.4127	0.41	0	17	0.02	1	U	R.SGFGDPIK.L 1111 1113
1211	415.2464	828.4782	828.4779	0.43	0	(16)	0.023	1	U	K.DLLPLMK.K 1209 1210 1212 1213 1214 1215 1216
1289	418.2208	834.4271	834.4269	0.24	1	8	0.14	1	U	R.NMKLDSK.T 1290
1455	423.2436	844.4727	844.4728	-0.14	0	19	0.013	1	U	K.DLLPLMK.K 1452 1453 1454 1456
1533	426.2180	850.4214	850.4218	-0.54	1	(6)	0.23	1	U	R.NMKLDSK.T 1534 1535
1814	439.7375	877.4605	877.4657	-5.97	0	8	0.17	1	U	K.AIENYIR.E
2338	463.2406	924.4666	924.4665	0.12	1	7	0.19	1	U	K.KTETYQR.A
2477	470.2430	938.4715	938.4723	-0.84	0	5	0.3	1	U	K.WGGVLDHR.S
2692	479.2935	956.5724	956.5728	-0.44	1	10	0.093	1	U	K.DLLPLMKK.T 2691
3381	505.7850	1009.5554	1009.5556	-0.17	0	13	0.051	1	U	R.LANLDHSLK.V 3380 3383 3384
3448	509.2650	1016.5153	1016.5152	0.18	1	3	0.53	1	U	K.WINRDTGR.R
3985	353.5476	1057.6209	1057.6205	0.36	1	(6)	0.23	1	U	K.TKDLLPLMK.K
3986	529.8178	1057.6209	1057.6205	0.40	1	17	0.019	1	U	K.TKDLLPLMK.K 3987 3988 3989
4064	355.5417	1063.6034	1063.6026	0.79	1	(9)	0.14	1	U	R.SNVYGVKGLK.V
4066	532.8093	1063.6040	1063.6026	1.32	1	18	0.014	1	U	R.SNVYGVKGLK.V 4065
4228	537.8151	1073.6156	1073.6154	0.11	1	(9)	0.14	1	U	K.TKDLLPLMK.K
4335	540.8245	1079.6345	1079.6339	0.56	0	12	0.071	1	U	R.TVPSKPLNPK.K 4331 4339
4337	360.8854	1079.6345	1079.6339	0.57	0	(8)	0.14	1	U	R.TVPSKPLNPK.K 4329 4330 4333 4334 4338 4340
4497	363.8731	1088.5976	1088.5978	-0.23	1	(14)	0.038	1	U	R.SGFGDPIKLR.A 4488 4489 4490 4492 4494 4495 4496 4498 [illegible]
4499	545.3062	1088.5977	1088.5978	-0.08	1	17	0.018	1	U	R.SGFGDPIKLR.A 4493 4501 4502 4504 4517 4532
5387	381.8839	1142.6300	1142.6295	0.40	1	6	0.23	1	U	K.VDKIIVEDGR.A
6379	604.8350	1207.6554	1207.6561	-0.58	1	16	0.028	1	U	K.LGTYEKTGLAR.F
6380	403.5593	1207.6561	1207.6561	0.00	1	(2)	0.68	1	U	K.LGTYEKTGLAR.F
7280	635.3670	1268.7194	1268.7201	-0.48	1	6	0.27	1	U	K.IIVEDGRAAAVR.T 7279
7282	423.9145	1268.7216	1268.7201	1.17	1	(4)	0.43	1	U	K.IIVEDGRAAAVR.T
7418	427.2471	1278.7194	1278.7197	-0.25	1	(11)	0.089	1	U	K.IVKWGGVLDHR.S
7419	640.3670	1278.7194	1278.7197	-0.19	1	24	0.0041	1	U	K.IVKWGGVLDHR.S
8254	670.3300	1338.6454	1338.6464	-0.79	1	(7)	0.18	1	U	R.DMAPMVWAYKK.S
8435	678.3268	1354.6391	1354.6413	-1.63	1	15	0.029	1	U	R.DMAPMVWAYKK.S
9675	723.3323	1444.6500	1444.6517	-1.21	0	26	0.0024	1	U	R.SDSAHAFVHSTMR.N 9677 9684
9678	362.1701	1444.6512	1444.6517	-0.40	0	(12)	0.058	1	U	R.SDSAHAFVHSTMR.N 9681
9680	482.5579	1444.6518	1444.6517	0.00	0	(11)	0.077	1	U	R.SDSAHAFVHSTMR.N 9679 9682 9683
9718	724.4399	1446.8652	1446.8671	-1.29	0	20	0.01	1	U	R.AAGVKPLVNLPGVGR.N 9721 9725
9722	483.2963	1446.8672	1446.8671	0.06	0	(7)	0.18	1	U	R.AAGVKPLVNLPGVGR.N 9720 9723
9935	366.1691	1460.6473	1460.6467	0.43	0	(14)	0.036	1	U	R.SDSAHAFVHSTMR.N 9936
10444	752.8647	1503.7149	1503.7140	0.64	0	8	0.16	1	U	R.NHDNLYLICNTK.V
12098	822.4059	1642.7972	1642.7991	-1.16	0	13	0.048	1	U	R.IKPQYESFDDFVR.G
12099	548.6075	1642.8008	1642.7991	1.02	0	(10)	0.1	1	U	R.IKPQYESFDDFVR.G
12348	834.8937	1667.7728	1667.7726	0.13	0	9	0.12	1	U	R.ACNNPDIHGFEGPIK.V
12430	838.3467	1674.6788	1674.6798	-0.59	0	23	0.005	1	U	R.GSASDYDDFQAEGWK.T 12431 12432 12433

13202	874.9196	1747.8246	1747.8278	-1.84	0	24	0.0043	1	U	K.TASFYTSNPSPHLNGR.R
13205	583.6165	1747.8276	1747.8278	-0.14	0	(15)	0.033	1	U	K.TASFYTSNPSPHLNGR.R 13203 13204
13569	594.2560	1779.7461	1779.7464	-0.15	0	(14)	0.042	1	U	R.NFQDHYCFFSPYR.I 13572
13570	890.8805	1779.7464	1779.7464	0.02	0	19	0.011	1	U	R.NFQDHYCFFSPYR.I 13571
14208	616.3099	1845.9078	1845.9043	1.90	1	10	0.098	1	U	R.NHDNLYLICNTKVDK.I
14449	936.0250	1870.0355	1870.0346	0.46	0	52	5.9e-006	1	U	K.QIVLSCGTISSPLVLQR.S 14447 14448
14450	624.3527	1870.0363	1870.0346	0.91	0	(7)	0.2	1	U	K.QIVLSCGTISSPLVLQR.S 14451
14610	628.9528	1883.8365	1883.8375	-0.55	0	9	0.14	1	U	K.YMTMFHFLEYPFSR.G
14796	952.9174	1903.8202	1903.8224	-1.19	1	25	0.0035	1	U	R.GSASDYDDFQAEGWKTK.D
14798	635.6144	1903.8215	1903.8224	-0.49	1	(4)	0.36	1	U	R.GSASDYDDFQAEGWKTK.D 14797
14802	476.9893	1903.9281	1903.9289	-0.44	1	5	0.3	1	U	K.TASFYTSNPSPHLNGRR.A
15573	983.4588	1964.9030	1964.9091	-3.08	0	33	0.00048	1	U	K.VSFGNYTYPVCQDFLR.A 15574 15575 15577 15581
15576	655.9769	1964.9090	1964.9091	-0.07	0	(22)	0.0057	1	U	K.VSFGNYTYPVCQDFLR.A
15965	667.0509	1998.1309	1998.1296	0.65	1	11	0.081	1	U	R.KQIVLSCGTISSPLVLQR.S 15964
18189	548.7728	2191.0620	2191.0658	-1.74	1	(1)	0.73	1	U	K.LDSKTASFYTSNPSPHLNGR.R
18190	731.3618	2191.0635	2191.0658	-1.06	1	19	0.013	1	U	K.LDSKTASFYTSNPSPHLNGR.R
19495	1153.0768	2304.1390	2304.1420	-1.30	0	31	0.00076	1	U	M.AIPEEFDILVLGGGSSGSCIAGR.L 19491 19492 19494
19498	769.0541	2304.1404	2304.1420	-0.69	0	(16)	0.028	1	U	M.AIPEEFDILVLGGGSSGSCIAGR.L 19496 19497
20302	1179.5669	2357.1192	2357.1330	-5.85	0	38	0.00014	1	U	R.AIVPCANVLGGGSSINFMMYTR.G
20303	786.7158	2357.1255	2357.1330	-3.21	0	(15)	0.032	1	U	R.AIVPCANVLGGGSSINFMMYTR.G
20441	1187.5609	2373.1073	2373.1279	-8.71	0	(30)	0.00096	1	U	R.AIVPCANVLGGGSSINFMMYTR.G 20445
20443	1187.5690	2373.1234	2373.1279	-1.92	0	(16)	0.024	1	U	R.AIVPCANVLGGGSSINFMMYTR.G
20444	792.0507	2373.1302	2373.1279	0.93	0	(20)	0.0092	1	U	R.AIVPCANVLGGGSSINFMMYTR.G
20526	597.0485	2384.1650	2384.1648	0.06	1	(4)	0.4	1	U	R.IKPQYESFDDFVRGDAEIQK.R 20530
20527	795.7291	2384.1656	2384.1648	0.31	1	18	0.014	1	U	R.IKPQYESFDDFVRGDAEIQK.R 20523 20525
20639	1195.5696	2389.1246	2389.1229	0.73	0	(2)	0.59	1	U	R.AIVPCANVLGGGSSINFMMYTR.G
20826	803.4010	2407.1812	2407.1808	0.14	0	(20)	0.0092	1	U	R.VFDQWYANGTGPLATNGIEAGVK.I 20828
20830	1204.6002	2407.1859	2407.1808	2.10	0	43	5.1e-005	1	U	R.VFDQWYANGTGPLATNGIEAGVK.I 20824 20825
20884	804.7089	2411.1048	2411.1063	-0.65	0	31	0.00077	1	U	K.IRPTPEELSQMDESFQEGYR.E 20885
21078	810.0394	2427.0965	2427.1012	-1.96	0	(23)	0.0053	1	U	K.IRPTPEELSQMDESFQEGYR.E
22069	629.5419	2514.1384	2514.1386	-0.11	0	(7)	0.2	1	U	R.MDHFAGEVTSHHPLFPYSSEAR.A 22067
22071	839.0536	2514.1389	2514.1386	0.12	0	(4)	0.36	1	U	R.MDHFAGEVTSHHPLFPYSSEAR.A 22068
22238	844.3841	2530.1304	2530.1336	-1.23	0	17	0.02	1	U	R.MDHFAGEVTSHHPLFPYSSEAR.A
22240	633.5407	2530.1337	2530.1336	0.07	0	(8)	0.17	1	U	R.MDHFAGEVTSHHPLFPYSSEAR.A 22239
22395	1271.6206	2541.2267	2541.2309	-1.66	0	70	9.2e-008	1	U	K.TATLVGEDLGYSGEALDMTVPQFK.L 22393 22394 22396 22398 [illegible]
22399	848.0843	2541.2310	2541.2309	0.06	0	(17)	0.018	1	U	K.TATLVGEDLGYSGEALDMTVPQFK.L 22397 22400
22625	1279.6155	2557.2164	2557.2258	-3.67	0	(18)	0.016	1	U	K.TATLVGEDLGYSGEALDMTVPQFK.L
22626	853.4156	2557.2249	2557.2258	-0.34	0	(8)	0.16	1	U	K.TATLVGEDLGYSGEALDMTVPQFK.L
22704	1282.6440	2563.2735	2563.2819	-3.28	1	(9)	0.13	1	U	K.RVFDQWYANGTGPLATNGIEAGVK.I
22706	855.4331	2563.2775	2563.2819	-1.73	1	37	0.0002	1	U	K.RVFDQWYANGTGPLATNGIEAGVK.I
24049	668.5664	2670.2365	2670.2397	-1.20	1	36	0.00028	1	U	R.RMDHFAGEVTSHHPLFPYSSEAR.A 24051 24052 24054 24056
24050	535.0547	2670.2371	2670.2397	-1.00	1	(21)	0.008	1	U	R.RMDHFAGEVTSHHPLFPYSSEAR.A 24053 24055 24057 24058 [illegible]
24180	672.5651	2686.2314	2686.2347	-1.22	1	(28)	0.0017	1	U	R.RMDHFAGEVTSHHPLFPYSSEAR.A 24182 24184
24181	538.2541	2686.2341	2686.2347	-0.22	1	(8)	0.16	1	U	R.RMDHFAGEVTSHHPLFPYSSEAR.A 24183 24185
24271	1348.6467	2695.2789	2695.2833	-1.64	0	12	0.058	1	U	K.VGDLSVCPDNVGCNTYTTALLIGEK.T
24272	899.4342	2695.2808	2695.2833	-0.95	0	(8)	0.15	1	U	K.VGDLSVCPDNVGCNTYTTALLIGEK.T
24295	675.3541	2697.3874	2697.3914	-1.50	0	(20)	0.0098	1	U	K.VGLIEAGENNLNNPWVYLPGIYPR.N
24298	1349.7025	2697.3905	2697.3914	-0.36	0	38	0.00015	1	U	K.VGLIEAGENNLNNPWVYLPGIYPR.N 24297
24299	900.1375	2697.3907	2697.3914	-0.28	0	(19)	0.013	1	U	K.VGLIEAGENNLNNPWVYLPGIYPR.N 24301 24302
24727	917.0788	2748.2146	2748.2126	0.72	0	(10)	0.097	1	U	R.GSIHITSPDPYAAPDFDPGFMNDER.D 24726
24728	1375.1183	2748.2220	2748.2126	3.43	0	12	0.057	1	U	R.GSIHITSPDPYAAPDFDPGFMNDER.D
24839	922.4083	2764.2032	2764.2075	-1.58	0	(9)	0.14	1	U	R.GSIHITSPDPYAAPDFDPGFMNDER.D
25134	938.0762	2811.2067	2811.2107	-1.44	0	36	0.00028	1	U	K.NEGHVTSNQVELHPDIEYDEEDDK.A
25135	703.8094	2811.2085	2811.2107	-0.81	0	(22)	0.0057	1	U	K.NEGHVTSNQVELHPDIEYDEEDDK.A
25955	745.8636	2979.4253	2979.4250	0.08	0	32	0.00061	1	U	R.ASESQGIPYVDDLEDLVTAHGAEHWLK.W 25958
25956	1490.7201	2979.4256	2979.4250	0.20	0	(12)	0.058	1	U	R.ASESQGIPYVDDLEDLVTAHGAEHWLK.W
25957	994.1494	2979.4264	2979.4250	0.46	0	(13)	0.051	1	U	R.ASESQGIPYVDDLEDLVTAHGAEHWLK.W
27206	1078.5341	3232.5804	3232.5850	-1.43	1	32	0.00066	1	U	K.TATLVGEDLGYSGEALDMTVPQFKLGTYEK.T 27207 27208 27209
27212	1617.3038	3232.5931	3232.5850	2.52	1	(4)	0.41	1	U	K.TATLVGEDLGYSGEALDMTVPQFKLGTYEK.T
27255	1083.8694	3248.5863	3248.5799	1.98	1	(32)	0.00068	1	U	K.TATLVGEDLGYSGEALDMTVPQFKLGTYEK.T
27509	824.9276	3295.6813	3295.6871	-1.74	1	(11)	0.079	1	U	M.AIPEEFDILVLGGGSSGSCIAGRLANLDHSLK.V
27510	1099.5700	3295.6880	3295.6871	0.29	1	21	0.0082	1	U	M.AIPEEFDILVLGGGSSGSCIAGRLANLDHSLK.V 27508
27772	1114.5536	3340.6389	3340.6398	-0.24	0	71	7.4e-008	1	U	R.ALEMDLETSNAYGGPLNLSAGLAHGSWTQPLK.K 27769 27770 27771 [illegible]
27778	836.1696	3340.6494	3340.6398	2.88	0	(24)	0.0041	1	U	R.ALEMDLETSNAYGGPLNLSAGLAHGSWTQPLK.K
27846	1119.8879	3356.6420	3356.6347	2.18	0	(56)	2.8e-006	1	U	R.ALEMDLETSNAYGGPLNLSAGLAHGSWTQPLK.K 27845
28458	1775.3712	3548.7279	3548.7324	-1.28	1	(3)	0.49	1	U	R.ASESQGIPYVDDLEDLVTAHGAEHWLKWINR.D
28460	888.1908	3548.7341	3548.7324	0.47	1	25	0.003	1	U	R.ASESQGIPYVDDLEDLVTAHGAEHWLKWINR.D
28461	710.7543	3548.7350	3548.7324	0.72	1	(16)	0.023	1	U	R.ASESQGIPYVDDLEDLVTAHGAEHWLKWINR.D 28462
28623	904.6755	3614.6728	3614.6711	0.47	1	9	0.12	1	U	R.ACNNPDIHGFEGPIKVSFGNYTYPVCQDFLR.A 28622
28624	1205.8984	3614.6735	3614.6711	0.65	1	(4)	0.36	1	U	R.ACNNPDIHGFEGPIKVSFGNYTYPVCQDFLR.A
28883	735.1392	3670.6594	3670.6659	-1.76	1	8	0.16	1	U	K.NEGHVTSNQVELHPDIEYDEEDDKAIENYIR.E
28985	1230.6528	3688.9367	3688.9365	0.04	1	35	0.00028	1	U	R.LANLDHSLKVGLIEAGENNLNNPWVYLPGIYPR.N
28986	923.2442	3688.9477	3688.9365	3.03	1	(34)	0.00043	1	U	R.LANLDHSLKVGLIEAGENNLNNPWVYLPGIYPR.N
29617	774.2003	3865.9649	3865.9672	-0.60	1	(20)	0.011	1	U	R.ALEMDLETSNAYGGPLNLSAGLAHGSWTQPLKKPTAK.N 29613 29614 [illegible]
29619	967.4996	3865.9694	3865.9672	0.57	1	25	0.0035	1	U	R.ALEMDLETSNAYGGPLNLSAGLAHGSWTQPLKKPTAK.N 29612 29615 [illegible]
29708	971.4903	3881.9321	3881.9621	-7.74	1	(20)	0.011	1	U	R.ALEMDLETSNAYGGPLNLSAGLAHGSWTQPLKKPTAK.N 29712
29710	777.4000	3881.9634	3881.9621	0.33	1	(13)	0.05	1	U	R.ALEMDLETSNAYGGPLNLSAGLAHGSWTQPLKKPTAK.N 29709

2. Dihydroxyacetone synthase variant 1 [Komagataella phaffii CBS 7435] **Mass:** 79350 **Score:** 1049 **Matches:** 242(107) **Sequences:** 61[illegible]

Dihydroxyacetone synthase variant 1 [Komagataella phaffii CBS 7435]

Query	Observed	Mr(expt)	Mr(calc)	ppm	Miss	Score	Expect	Rank	Unique	Peptide

99	352.1925	702.3704	702.3701	0.47	0	22	0.0058	1	U	K.AIDWAK.A 93 94 95 96 97 98
255	362.6944	723.3742	723.3744	-0.26	0	18	0.016	1	U	K.FWFPK.E 254 256 257
386	373.2262	744.4378	744.4382	-0.41	0	25	0.0029	1	U	R.DVATIVK.A 385 387 388 389 391 392
697	393.2498	784.4851	784.4919	-8.60	1	5	0.32	1	U	R.LLRQQK.G 698
879	399.7450	797.4755	797.4759	-0.52	1	6	0.23	1	U	R.VRGELPK.N 877 881
1058	408.2080	814.4015	814.4014	0.12	0	16	0.027	1	U	K.YFFNPK.L
1095	409.7282	817.4418	817.4406	1.48	1	9	0.12	1	U	K.TSAQGAKR.G 1094
1146	412.2316	822.4486	822.4487	-0.15	0	16	0.023	1	U	K.LVDSYVK.E 1147
1922	443.2797	884.5448	884.5443	0.57	1	16	0.028	1	U	R.EIVRALGK.N 1921
2000	449.2448	896.4751	896.4756	-0.58	0	13	0.05	1	U	R.YIEFGIR.E 1993 1999 2001
2086	452.2518	902.4891	902.4895	-0.44	0	10	0.098	1	U	R.MAALQELK.A 2089
2283	460.2500	918.4855	918.4844	1.18	0	(3)	0.47	1	U	R.MAALQELK.A
2321	462.7320	923.4494	923.4501	-0.80	0	13	0.051	1	U	K.YGFNPAQK.F 2319 2320 2322 2324
2393	466.2695	930.5245	930.5247	-0.16	1	8	0.17	1	U	R.TSAREIVR.A 2390 2391 2392 2394 2398
2626	476.2792	950.5438	950.5437	0.12	1	15	0.032	1	U	K.KLVDSYVK.E
3251	500.2720	998.5294	998.5297	-0.29	1	9	0.14	1	U	R.GELPKNWR.T
3311	502.7397	1003.4649	1003.4651	-0.20	0	4	0.42	1	U	K.YFGYTPEK.I 3308 3309 3310
3340	503.7610	1005.5075	1005.5066	0.89	0	6	0.24	1	U	R.VLSFPCQR.L 3339
3857	525.2795	1048.5445	1048.5441	0.44	0	19	0.014	1	U	K.SLPVEDVYK.Y 3855
3865	525.3029	1048.5912	1048.5917	-0.50	0	4	0.4	1	U	R.VVQYVNSIK.A 3867
4167	536.7567	1071.4989	1071.4985	0.32	0	(14)	0.037	1	U	R.HEVDQYPGK.T 4170
4168	358.1736	1071.4990	1071.4985	0.45	0	18	0.016	1	U	R.HEVDQYPGK.T
4454	363.5291	1087.5656	1087.5662	-0.55	1	(6)	0.28	1	U	K.GDELVKNWK.K
4456	544.7905	1087.5665	1087.5662	0.29	1	6	0.27	1	U	K.GDELVKNWK.K
5610	577.3048	1152.5949	1152.5927	1.91	1	12	0.061	1	U	K.TKYGFNPAQK.F 5608
6571	407.5508	1219.6307	1219.6309	-0.19	1	13	0.045	1	U	R.EGQEFLSRVR.G
7304	636.3168	1270.6190	1270.6194	-0.29	0	5	0.3	1	U	R.LFDEQSITYR.R 7305 7307
8641	456.9099	1367.7079	1367.7085	-0.43	1	(20)	0.009	1	U	K.LVDSYVKEYPR.E 8643 8644
8642	684.8613	1367.7080	1367.7085	-0.37	1	33	0.00048	1	U	K.LVDSYVKEYPR.E 8640
8669	686.3016	1370.5887	1370.5891	-0.28	0	12	0.068	1	U	K.YAPNDPDYFNR.D 8668
9080	466.9278	1397.7616	1397.7626	-0.77	0	(0)	0.95	1	U	K.AETERPTLINVR.T
9081	699.8884	1397.7622	1397.7626	-0.33	0	4	0.37	1	U	K.AETERPTLINVR.T
9414	712.8298	1423.6450	1423.6442	0.56	0	35	0.00033	1	U	R.YATAGYTMNTFGK.S 9413
9467	714.3680	1426.7214	1426.7205	0.67	1	7	0.21	1	U	R.LFDEQSITYRR.S 9464
9498	715.4050	1428.7955	1428.7977	-1.50	1	18	0.016	1	U	R.DVATIVKAIDWAK.A 9499
9500	477.2736	1428.7991	1428.7977	1.03	1	(9)	0.11	1	U	R.DVATIVKAIDWAK.A
9630	720.8254	1439.6363	1439.6391	-1.94	0	(33)	0.00051	1	U	R.YATAGYTMNTFGK.S 9631
9647	722.3606	1442.7066	1442.7082	-1.05	0	(10)	0.097	1	U	K.EVYDFFAEKPAK.G
9648	481.9099	1442.7079	1442.7082	-0.18	0	11	0.083	1	U	K.EVYDFFAEKPAK.G
9917	487.2462	1458.7169	1458.7143	1.79	1	14	0.036	1	U	K.YFGYTPEKIGER.V
10452	502.2875	1503.8407	1503.8409	-0.14	1	(12)	0.059	1	U	K.IGERVVQYVNSIK.A 10451
10454	752.9281	1503.8416	1503.8409	0.49	1	16	0.026	1	U	K.IGERVVQYVNSIK.A 10453
10830	765.8559	1529.6972	1529.6933	2.58	0	18	0.015	1	U	K.LQTFCGLGGDYSGR.Y 10829
11176	780.9061	1559.7976	1559.7984	-0.50	0	(7)	0.22	1	U	K.ASPQILYEFHDLK.G
11177	520.9401	1559.7985	1559.7984	0.10	0	9	0.14	1	U	K.ASPQILYEFHDLK.G
11469	793.8963	1585.7780	1585.7849	-4.30	0	15	0.031	1	U	R.AQNWNVIDIVDGSR.D 11470 11472 11473 11474 11475 11476
11471	529.6017	1585.7832	1585.7849	-1.04	0	(9)	0.12	1	U	R.AQNWNVIDIVDGSR.D
11650	534.6102	1600.8087	1600.8097	-0.62	0	(0)	0.91	1	U	K.AVSYNDDIHDLVIK.T
11652	801.4137	1600.8128	1600.8097	1.98	0	7	0.18	1	U	K.AVSYNDDIHDLVIK.T
11938	543.9456	1628.8149	1628.8140	0.54	1	6	0.25	1	U	K.YGFNPAQKFWFPK.E
12089	821.8663	1641.7180	1641.7172	0.50	1	(9)	0.12	1	U	K.YAPNDPDYFNRDR.F
12090	548.2473	1641.7199	1641.7172	1.68	1	12	0.069	1	U	K.YAPNDPDYFNRDR.F 12088
12859	572.6180	1714.8321	1714.8275	2.72	1	3	0.5	1	U	R.HEVDQYPGKTSAQGAK.R
12886	859.4419	1716.8692	1716.8683	0.57	0	31	0.00078	1	U	R.TYIPQDKPTEPTATR.T 12883 12889
12893	573.2986	1716.8741	1716.8683	3.42	0	(12)	0.062	1	U	R.TYIPQDKPTEPTATR.T 12885 12887 12888 12895 12896
13227	583.9399	1748.7978	1748.7974	0.25	0	6	0.25	1	U	R.EHSMCAIANGLAAYNK.G
15025	641.2899	1920.8479	1920.8465	0.77	1	1	0.75	1	U	K.YQMKYAPNDPDYFNR.D
15629	493.5230	1970.0628	1970.0625	0.15	1	5	0.33	1	U	K.ASPQILYEFHDLKGKPK.H
15631	657.6953	1970.0641	1970.0625	0.81	1	(2)	0.6	1	U	K.ASPQILYEFHDLKGKPK.H
16022	669.3471	2005.0195	2005.0269	-3.68	1	(16)	0.024	1	U	K.AVSYNDDIHDLVIKTFR.C
16024	1003.5192	2005.0238	2005.0269	-1.55	1	33	0.0005	1	U	K.AVSYNDDIHDLVIKTFR.C
16025	502.2636	2005.0252	2005.0269	-0.83	1	(17)	0.021	1	U	K.AVSYNDDIHDLVIKTFR.C
16359	1018.0057	2033.9968	2033.9986	-0.88	1	29	0.0012	1	U	K.SLPVEDVYKYFGYTPEK.I 16356 16362 16364
16366	679.0085	2034.0036	2033.9986	2.47	1	(16)	0.026	1	U	K.SLPVEDVYKYFGYTPEK.I 16358 16363 16365
16773	695.0478	2082.1215	2082.1221	-0.29	1	(0)	0.94	1	U	K.AIDWAKAETERPTLINVR.T
16775	1042.0686	2082.1226	2082.1221	0.24	1	12	0.056	1	U	K.AIDWAKAETERPTLINVR.T
17318	534.0286	2132.0851	2132.0862	-0.49	1	1	0.73	1	U	R.TYIPQDKPTEPTATRTSAR.E
17321	711.7035	2132.0888	2132.0862	1.24	1	(1)	0.85	1	U	R.TYIPQDKPTEPTATRTSAR.E
17508	717.0285	2148.0637	2148.0720	-3.89	1	17	0.022	1	U	K.FWFPKEVYDFFAEKPAK.G
17950	725.3706	2173.0900	2173.0916	-0.73	1	11	0.072	1	U	K.NWRTYIPQDKPTEPTATR.T
19603	771.7444	2312.2113	2312.2125	-0.50	1	(35)	0.00034	1	U	R.AQNWNVIDIVDGSRDVATIVK.A 19599 19600 19602 19604 19607 1
19606	1157.1140	2312.2135	2312.2125	0.43	1	42	6.8e-005	1	U	R.AQNWNVIDIVDGSRDVATIVK.A 19598 19601
22428	1272.1499	2542.2852	2542.2970	-4.63	0	25	0.003	1	U	K.GTFLPITSTFYMFYLYAAPALR.M
23939	887.7503	2660.2291	2660.2327	-1.37	0	22	0.0065	1	U	R.TEIGQDSAFGNHHAAHGSALGEEGIR.E
23940	666.0647	2660.2297	2660.2327	-1.15	0	(21)	0.0087	1	U	R.TEIGQDSAFGNHHAAHGSALGEEGIR.E
23941	533.0544	2660.2355	2660.2327	1.04	0	(5)	0.3	1	U	R.TEIGQDSAFGNHHAAHGSALGEEGIR.E
26129	1004.1650	3009.4733	3009.4641	3.05	0	9	0.13	1	U	R.GGYVVEDCEGKPDVQLIGTGSELEFAIK.T
26173	753.6393	3010.5283	3010.5261	0.72	0	(45)	2.9e-005	1	U	K.AIHIATHDSIGAGEDGPTHQPIALSSLFR.A 26143 26151 26152 2615
26174	603.1130	3010.5285	3010.5261	0.81	0	48	1.7e-005	1	U	K.AIHIATHDSIGAGEDGPTHQPIALSSLFR.A 26145 26147 26148 2614
26183	1004.5179	3010.5320	3010.5261	1.96	0	(23)	0.0052	1	U	K.AIHIATHDSIGAGEDGPTHQPIALSSLFR.A 26144 26146 26155
26330	758.6197	3030.4497	3030.4543	-1.55	1	33	0.0005	1	U	R.TEIGQDSAFGNHHAAHGSALGEEGIRELK.T
26331	607.0973	3030.4501	3030.4543	-1.41	1	(7)	0.18	1	U	R.TEIGQDSAFGNHHAAHGSALGEEGIRELK.T 26335 26340

26333	1011.1590	3030.4552	3030.4543	0.27	1	(31)	0.00079	1	U	R.TEIGQDSAFGNHHAAHGSALGEEGIRELK.T	26336
26334	506.0832	3030.4553	3030.4543	0.32	1	(2)	0.68	1	U	R.TEIGQDSAFGNHHAAHGSALGEEGIRELK.T	26332
27749	835.4202	3337.6516	3337.6500	0.46	1	21	0.0078	1	U	R.GGYVVEDCEGKPDVQLIGTGSELEFAIKTAR.L	27748 27750
29757	780.0090	3895.0085	3895.0050	0.89	1	19	0.012	1	U	R.MAALQELKAIHIATHDSIGAGEDGPTHQPIALSSLFR.A	
29758	650.1765	3895.0150	3895.0050	2.57	1	(14)	0.042	1	U	R.MAALQELKAIHIATHDSIGAGEDGPTHQPIALSSLFR.A	29754
30357	674.3356	4039.9701	4039.9848	-3.64	1	1	0.72	1	U	K.AETERPTLINVRTEIGQDSAFGNHHAAHGSALGEEGIR.E	30361
30405	674.5054	4040.9886	4041.0016	-3.22	0	4	0.41	1	U	R.AMPNFYYMRPADATEVAALFEVAVELEHSTLLSLSR.H	30399 30402
32681	1219.3398	4873.3303	4873.3617	-6.46	0	(32)	0.00063	1	U	K.QLQSYHSSDYHSLTPGHPEIENPAVEVTTGPLGQGISNAVGMAIGSK.N	326[illegible]
32688	975.6799	4873.3633	4873.3617	0.32	0	(32)	0.00069	1	U	K.QLQSYHSSDYHSLTPGHPEIENPAVEVTTGPLGQGISNAVGMAIGSK.N	326[illegible]
32772	978.8781	4889.3542	4889.3566	-0.51	0	34	0.00043	1	U	K.QLQSYHSSDYHSLTPGHPEIENPAVEVTTGPLGQGISNAVGMAIGSK.N	327[illegible]
32773	1223.3464	4889.3567	4889.3566	0.00	0	(23)	0.0051	1	U	K.QLQSYHSSDYHSLTPGHPEIENPAVEVTTGPLGQGISNAVGMAIGSK.N	327[illegible]

Mascot: http://www.matrixscience.com/

Supporting information. Confirmation of presence of DHAS and AOX in protein bands of GS115 (Fig. 3A) by mass spectrometry. Protein bands of interest were excised from the gel and subjected to in-gel trypsin digestion. MALDI- TOF was performed in an HCT Ultra PTM Discovery System (ETD II, Bruker Daltonics) with an 1100 series HPLC (Agilent). For the identification of proteins, the obtained “peak list” was searched against the NCBI nonredundant database using Mascot protein mass fingerprint software. Molecular weight of DHAS and AOX was 79350 and 74812 daltons respectively.
