# Supplementary material for: Synthetic transcription factors establish the function of nine amino acid transactivation domains of Komagataella phaffii Mxr1
Source: J Biol Chem. 2025 Jan 22;301(3):108211. doi: 10.1016/j.jbc.2025.108211 (PMC11872449; doi:10.1016/j.jbc.2025.108211)
Supplement: Supporting Information Figures [file mmc2.docx]

**Supporting Information Figures**

**Manuscript Title: Synthetic transcription factors establish the function of nine amino**

**acid transactivation domains of *Komagataella phaffii* Mxr1**

**Authors: Prachi Priya, Vedanth Bellad Shivashankar and Pundi N Rangarajan**

**Content**

**Figure S1. 3D structure prediction of** **Mxr1ZF-TAD A^3x^** **by Alphafold2.**

**Figure S2. Nucleotide sequence of synthetic genes encoding 3 copies of putative**

**9aaTADs (TAD C-V^3X^) of Mxr1.**

**Figure S3. Analysis of subcellular localization of the Mxr1ZF-TAD A^3x^and Mxr1ZF**

**TAD C-V^3x^ by fluorescence microscopy.**

**Figure S4. Functional 9aaTADs of *K. phaffii* Mxr1.**

**Figure S5. 3D structure prediction of Mxr1 by Alphafold3.**

**Figure S1 Prachi Priya et al.,**


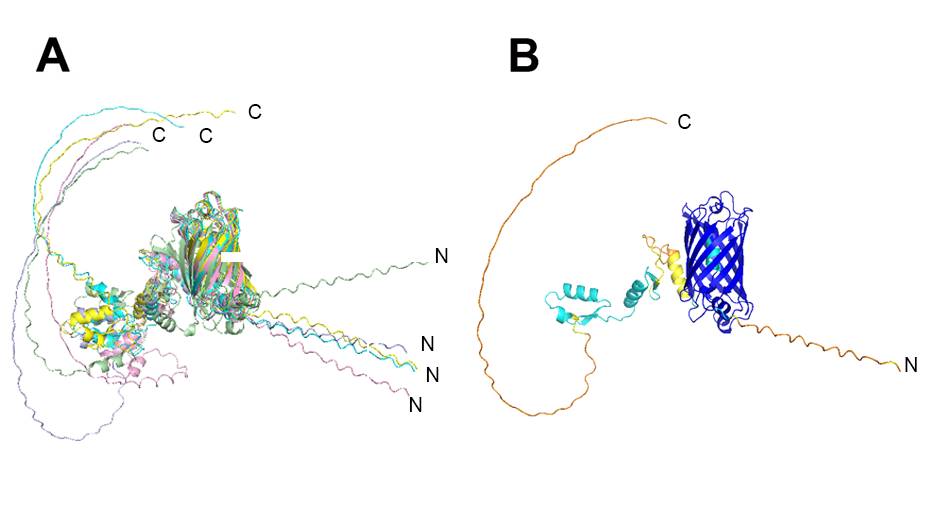


**Figure S1. 3D structure prediction of Mxr1 by Alphafold3. A.** Overlay of five best models of Mxr1ZF-TAD A^3x^ predicted by Alphafold2. **B.** AlphaFold2 predicted structure is shown in colors of pLDDT values. Regions with high accuracy (pLDDT > 90) in Blue, regions with moderate accuracy (pLDDT < 90 and >= 70) in Cyan, regions with low accuracy (pLDDT < 70 and >= 50) in Yellow, regions with very low accuracy (pLDDT < 50) in Orange. N-terminus (N) and C-terminus (C) are indicated.

**Figure S2 Prachi Priya et al.,**

TAD C^3x^:

TAD D^3x^:

TAD E^3x^:

TAD F^3x^:

TAD G^3x^:

TAD H^3x^:

TAD I^3x^:

TAD J^3x^:

TAD K^3x^:

TAD L^3x^:

TAD M^3x^:

TAD N^3x^:

TAD O^3x^:

TAD P^3x^:

TAD Q^3x^:

TAD R^3x^:

TAD S^3x^:

TAD T^3x^:

TAD U^3x^:

TAD V^3x^:

**Figure S2.** **Nucleotide sequence of synthetic genes encoding 3 copies of putative 9aaTADs (TAD C-V^3X^) of Mxr1.** TADs are separated by 10 amino acid linkers (L1, L2). Synthetic gene fragments obtained from Genescript, USA were digested with XhoI and NotI and cloned into XhoI-NotI digested pMxr1ZF vector (See Fig. 5A). Amino acid sequence deduced from the nucleotide sequence is shown. TAD B could not be synthesized due to technical difficulties and hence was not included in this study.

**Figure S3 Prachi Priya et al.,**


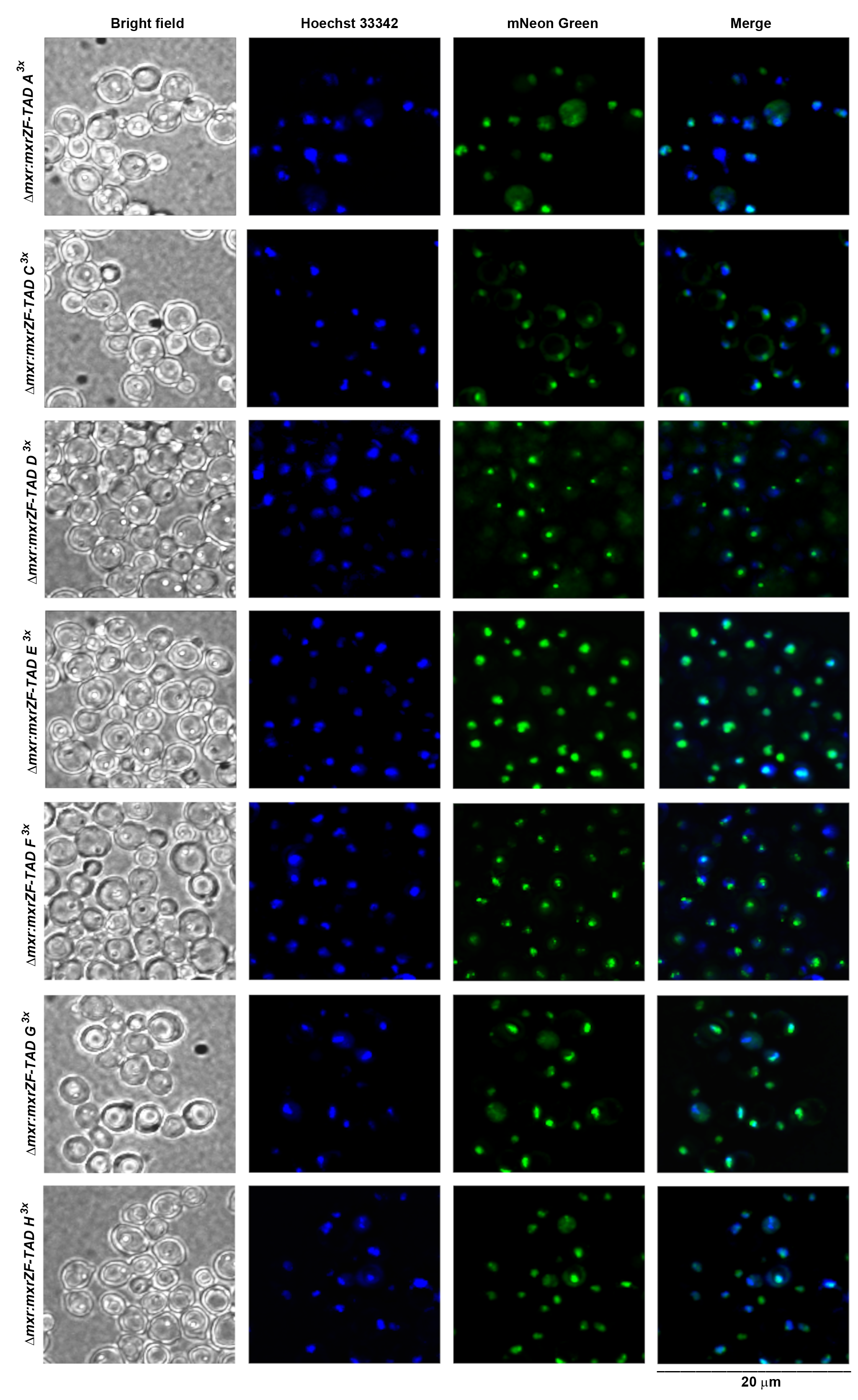


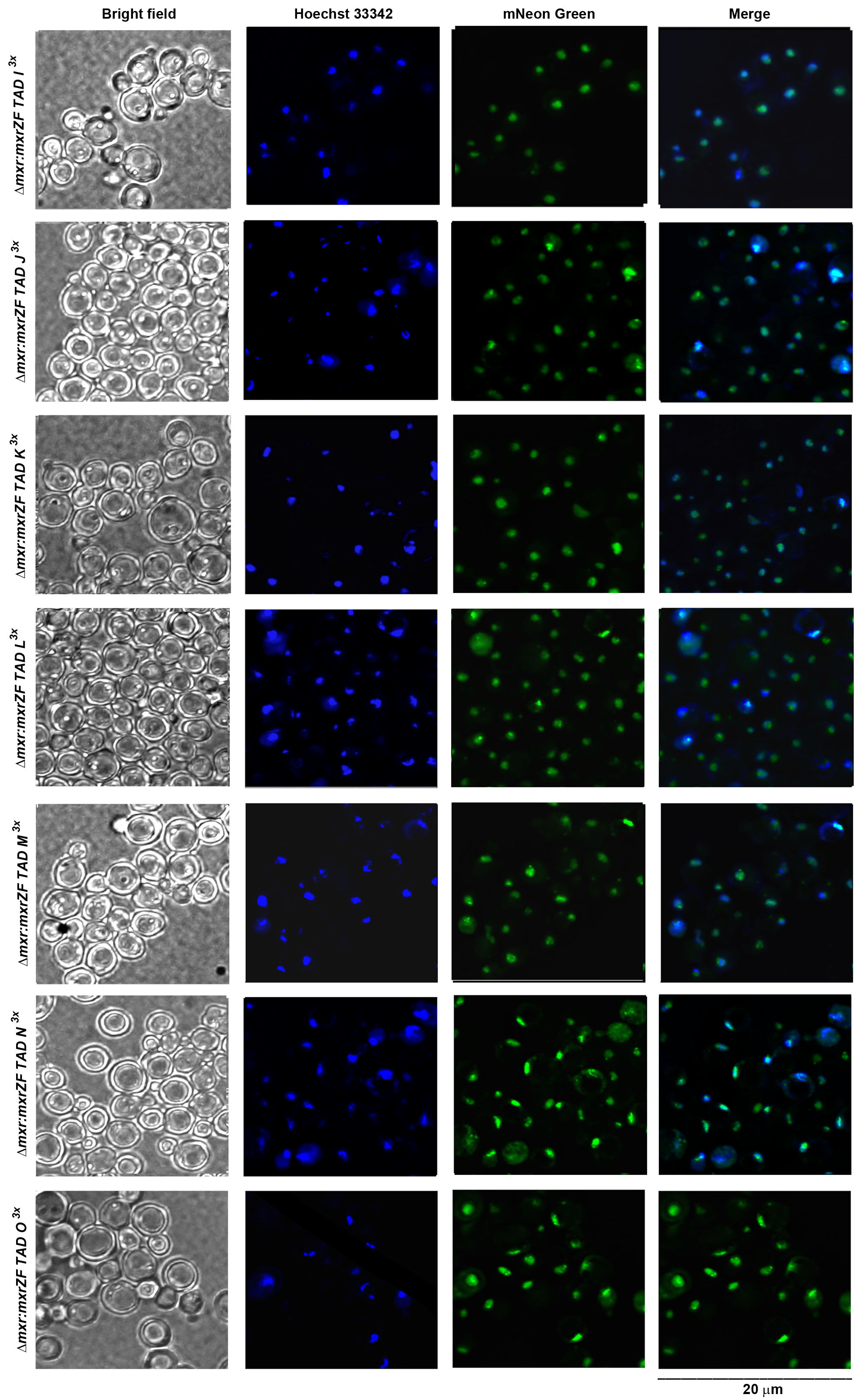


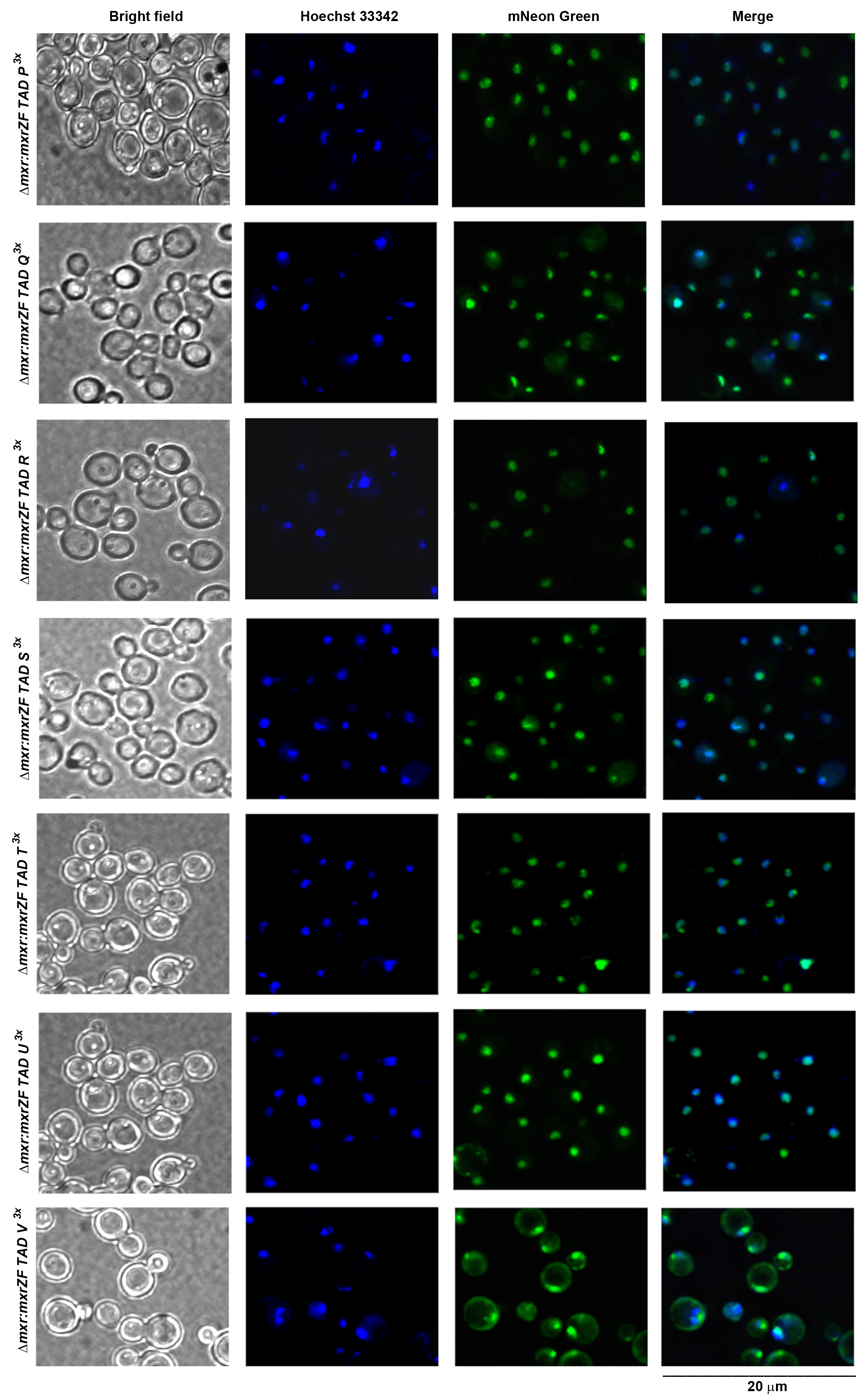
**Figure S3.** **Analysis of subcellular localization of the Mxr1ZF-TAD A^3x^and Mxr1ZF-TAD C-V^3x^ by fluorescence microscopy.** Hoechst 33342 stains the nucleus. Cells were cultured in YNBE for 3 h.

**Figure S4 Prachi Priya et al.,**


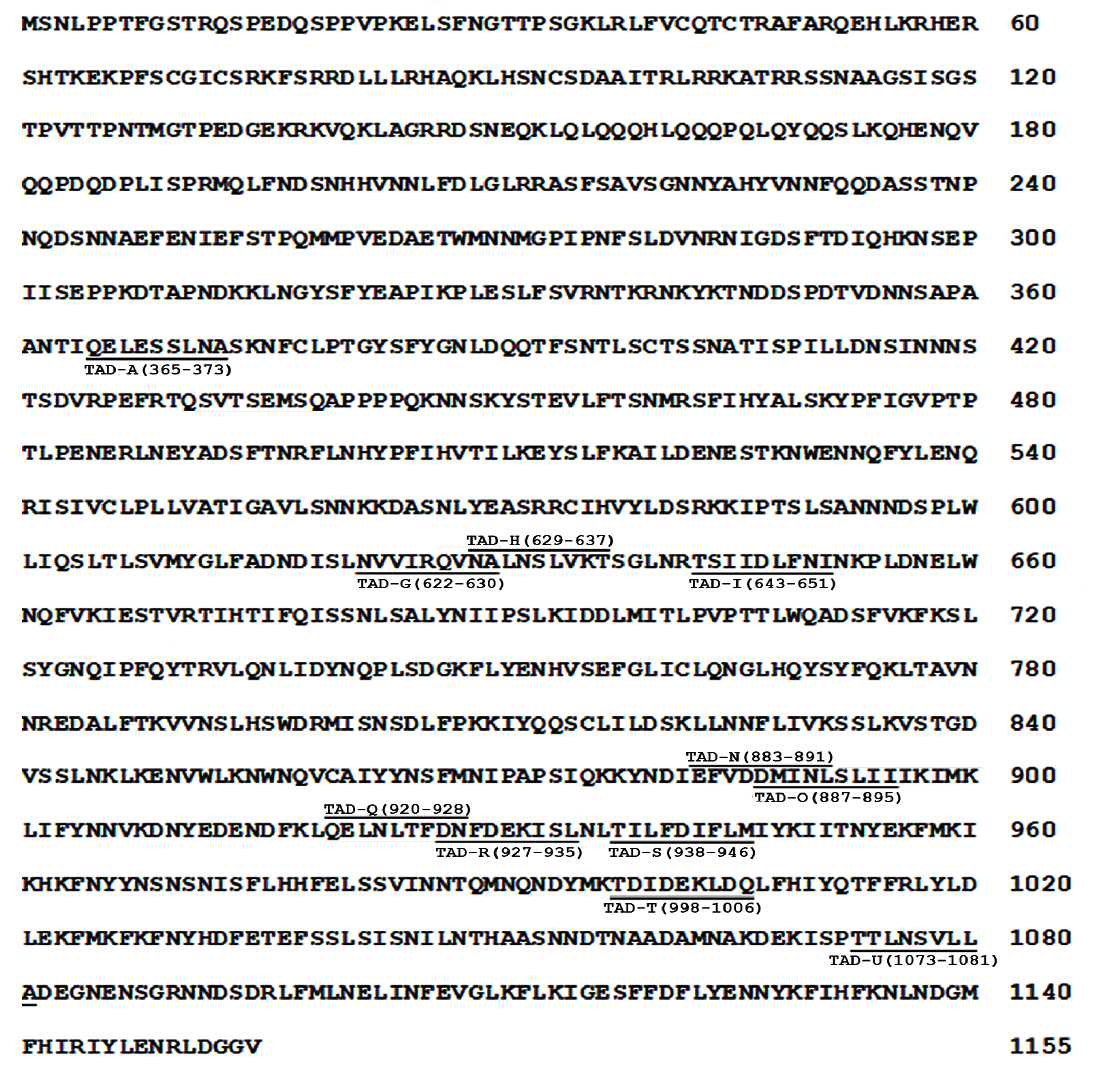


**Figure S4.** **Functional 9aaTADs of *K. phaffii* Mxr1.** Amino acid sequence of *K. phaffii* Mxr1 depicting the position of functional 9aaTADs (underlined).

**Figure S5 Prachi Priya et al.,**


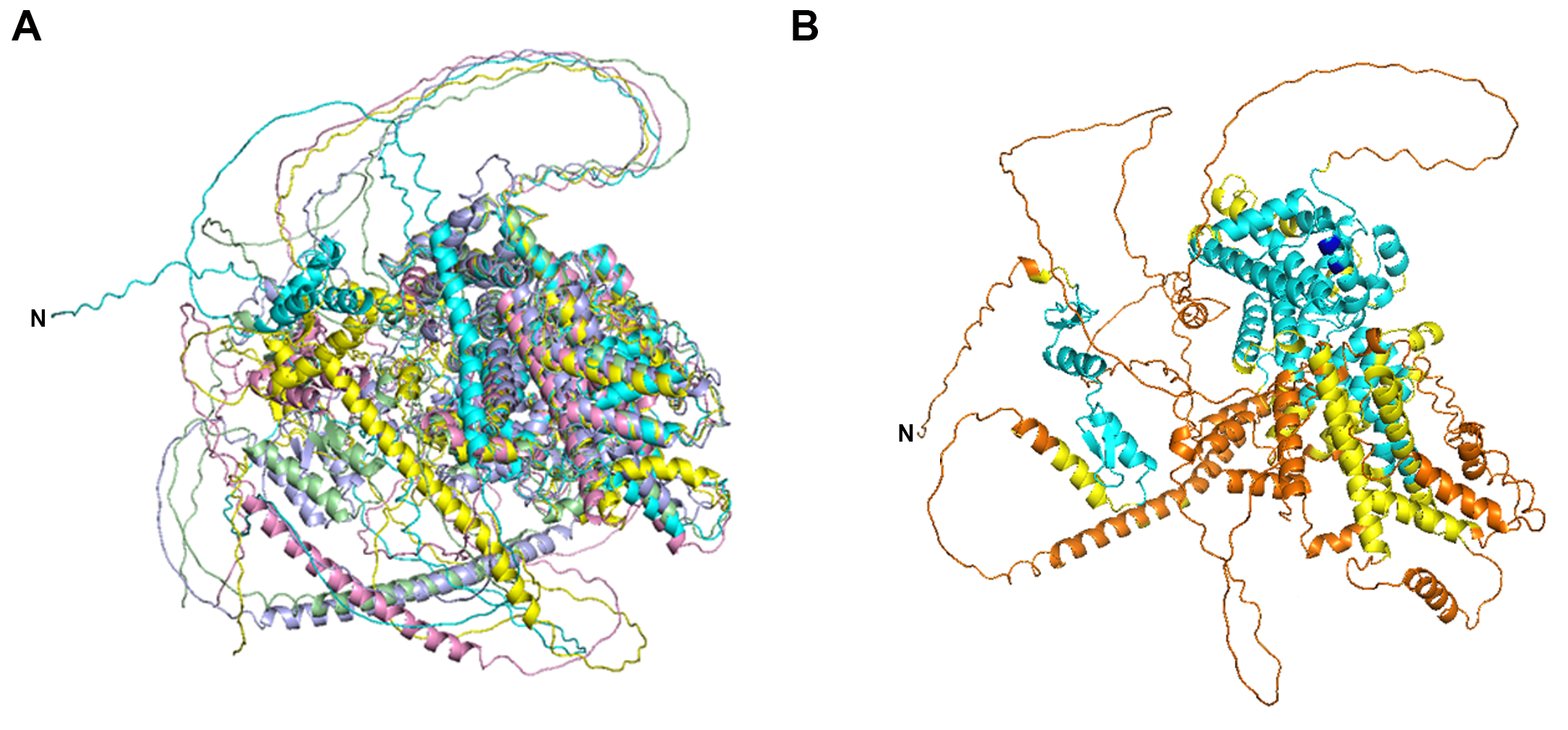


**Figure S5. 3D structure prediction of Mxr1 by Alphafold3. A.** Overlay of five best models of Mxr1 predicted by Alphafold3. **B.** AlphaFold3 predicted structure is shown in colors of pLDDT values. Regions with high accuracy (pLDDT > 90) in Blue, regions with moderate accuracy (pLDDT < 90 and >= 70) in Cyan, regions with low accuracy (pLDDT < 70 and >= 50) in Yellow, regions with very low accuracy (pLDDT < 50) in Orange. N-terminus (N) is indicated.
